# Systematic review of ethnomedicine, phytochemistry, and pharmacology of *Cyperi Rhizoma*

**DOI:** 10.3389/fphar.2022.965902

**Published:** 2022-10-07

**Authors:** Fengyou Wang, Shihao Zhang, Jiaxu Zhang, Fu Yuan

**Affiliations:** Key Laboratory of Chinese Materia Medica, Ministry of Education, Heilongjiang University of Chinese Medicine, Harbin, China

**Keywords:** Cyperi rhizoma, chemical constituents, pharmacological effects, traditional applications, processing

## Abstract

*Cyperi Rhizoma* (CR) is the dry rhizome of *Cyperus rotundus* L., a Cyperaceae plant. It has a long history of clinical medication and is known as the “holy medicine” of gynecology. CR smells sweet and bitter. It has the effect of soothing the liver and relieving depression, regulating qi, regulating meridian and relieving pain. It can be used to treat liver qi stagnation, chest pain, spleen and stomach qi stagnation, hernia pain, irregular menstruation and other diseases. At present, the main chemical constituents isolated from CR are volatile oil, flavonoids and terpenes. Modern pharmacological studies have shown that CR has a wide range of pharmacological activities, including antidepressant, hypoglycemic, antioxidant, anti-inflammatory, antipyretic and analgesic effects. In this paper, the botany, traditional application, phytochemistry, pharmacological effects, processing and other aspects of CR are reviewed. At the same time, the shortcomings of current research of CR are discussed in depth, and the possible solutions are put forward in order to find a breakthrough point for future research of CR.

## 1 Introduction

Cyperaceae plants *Cyperus rotundus* L. is widely distributed in subtropical and tropical regions of the world, which invade crops and have a destructive impact on agricultural production (Stierle et al., 1991). Although it was found as a harmful substance in cultivated land, it has several beneficial medicinal values since ancient times. *Cyperi Rhizoma* (CR) is the dry rhizome of *Cyperus rotundus L*. In Western Asia, CR is used in the treatment of leprosy, thirst, fever and hematological diseases in Western Asian traditional medicine (Tsoyi et al., 2011; Ito et al., 2012). In Egyptian folk medicine, CR is used for expelling evil, strengthening yang, diuresis, sedation, expelling wind, stimulation and nourishing, and for the treatment of renal colic and stomach pain. In India, they are also recommended for the treatment of diabetes, arthritis, diarrhoea, dysentery, leprosy, bronchitis, amenorrhea, dysmenorrhea, fever, arthritis and blood diseases (Babiaka et al., 2021; Taheri et al., 2021).

**FIGURE 1:**
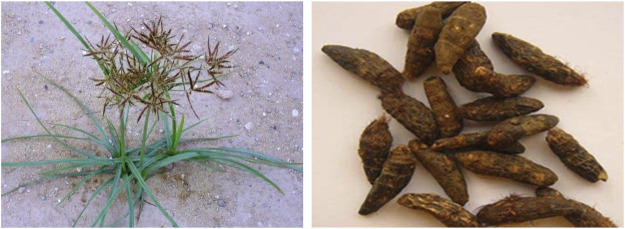
Cyperi rhizoma.

As a traditional Chinese medicine, Chinese name Xiangfu, also known as the root of *Cyperus rotundus* L., xiangfuzi, Xiangfumi, started in Tao Hongjing’s Famous Medical Record (名医别录), listed as medium-grade drug, sweet, slightly cold, non-toxic. CR has the effects of soothing liver and relieving depression, regulating qi, regulating menstruation and relieving pain, which can be used to treat liver qi stagnation, chest pain, spleen and stomach qi stagnation, hernia pain, irregular menstruation and other diseases. As a plant widely distributed around the world, the rhizome of *Cyperus rotundus* L. has been used for perfume and spice and Ayurvedic therapy in Arab countries and Africa and India for centuries (Sharma and Gupta, 2007). The leaves of *Cyperus rotundus L*. are widely used to flavor food, especially in the Middle East and Southeast Asia. At present, phytochemical studies have shown that CR contains volatile oil, flavonoids, triterpenes, sterols, alkaloids, sugars, trace elements and other components. Modern pharmacological studies have shown that CR has antioxidant effect, anti-inflammatory effect, antidepressant effect, antipyretic analgesic effect, antitumor effect, hypoglycemic effect, antibacterial effect and other effects, which are commonly used in the treatment of nervous system, cardiovascular system, digestive system, uterus and other diseases in clinic (Xiao-Ting et al., 2012; Chen et al., 2017; Dong-Bao et al., 2017). The picture of CR is shown in Figure 1.

In this review, we conducted a comprehensive search of the following databases: CNKI、VIP、PubMed、Web of Science、ResearchGate、sciencedabase、SciFinder、Science Direct、Wiley Online、Google Scholar、Classics of Traditional Chinese Medicine (Encyclopedia of traditional Chinese medicine (中医百科全书)、Chinese medical classics (中医典籍)、Dictionary of traditional Chinese medicine (中医大辞典)) and PhD, Master’s degree papers and other databases. We searched for studies published between 1990 and 2022. Key words: *Cyperi Rhizoma*, bioactive compounds, pharmacological activities. We delete duplicate papers, and then screen data to exclude irrelevant paper studies. In this paper, the latest research progress in chemical composition, biological activity and pharmacological action of such natural substances is comprehensively, deeply and systematically expounded. The safety and toxicity of CR extract were also considered in the manuscript. This paper aims to further understand CR and provide reference for the study of biological activity and clinical application of it, in order to provide scientific basis for the rational development of new drugs. Compared with other reviews, this study provides a more comprehensive view of the botanical, phytochemical and pharmacological effects and complements the elaboration of traditional applications and processing. At the same time, the limitations of current CR research were discussed, and the future research direction of CR was prospected, so as to provide scientific basis for the development of CR with greater therapeutic potential and the rational development of new drugs.

## 2 Botany

According to the dictionary of traditional Chinese medicine (中药大辞典), CR is the dried root of *Cyperus rotundus* L. Spring, summer and autumn can be harvested, usually in the autumn digging rhizomes, with fire to the fibrous roots and leaves, into boiling water for a moment, or evaporation in the evaporation cage to remove drying. Then put it back and forth in the bamboo cage; the dust and whiskers were removed with bamboo sieve, and then the light CR formed. There are also without fire, the rhizome into a sack rubbed after drying. It also uses stone milling to remove the fur, which is called Xiangfumi. Dry rhizomes often fusiform, sometimes slightly curved. 1.5–4.5 cm long, 5–10 mm in diam. Surface brown or dark brown, with longitudinal wrinkles and several uplifting links, with brown hairy scales and residual root marks on nodes; if the hair is removed, the appearance is smooth and the link is not obvious. Hard, after cooking section brown yellow and slightly purple red, cuticle; the cross section of sunburners was white and pink, and the surrounding and center layers were obvious, and the center color was slightly dark. CR, bitter taste. With a large, brown mattress, solid quality, strong aroma for the best. Mainly produced in Shandong, Zhejiang, Hunan, Henan, other areas also have production. Among them, Dongxiangfu was produced in Shandong and Nanxiangfu was produced in Zhejiang, with better quality.

CR grows in the wet places of grassland, cultivated land and roadside water. It enjoys warm and humid climate and humid environment. It is cold-resistant and suitable for growth in loose sandy loam. Domestic mainly distributed in Shandong, Zhejiang, Hunan, Henan and other places, abroad widely distributed around the world.

Because CR is one of the commonly used traditional Chinese medicines, there are two counterfeits in the market, Cyperus stoloniferus and rhizoma anemones raddeanae. The characters and sources are shown in Supplementary Table A1. At the same time, the three traits are extremely similar, but the efficacy is different. In clinical use must be carefully identified to avoid the impact of clinical medication safety and efficacy.

## 3 Clinical applications

### 3.1 Traditional application

CR, as a traditional Chinese medicine of medicinal use and has been recorded in many books (Ning-Ping et al., 2015). Xiangfu is also known as Quetouxiang, sedge root, Xiangfuzi, Leigongtou, Xiangfumi, etc. Xiangfu medicine was first recorded in Tao Hongjing’s Ming Yi Bie Lu (名医别录) (Wei and Jin Dynasties: A.D. 220–450) in the name of sedge root. It was first recorded that CR was listed as a medium-grade, sweet, slightly cold, non-toxic. Mainly used to remove heat in the chest, take a long time can fill the air, promote eyebrows and whiskers growth. The interpretation of Bencao Jing was first listed as “Xiang Fu” in the Ministry of grass, and later generations mostly used “Xiang Fu” as its name. "Guangya” contained: “ground hair.” Xiangfu is also known as “ground hair”, because it is long underground, circumferentially hairy. “Xinxiu Bencao” records: “This grass, root name Xiangzi, a finch head Xiang... Stems, leaves are like three edges, root like aconite, hairy circumferential turns...... Jingxiang. People called it sedge root, and it was used in harmony with incense.” It can be seen that its root gas incense and like aconite and get the name. The Compendium of Materia Medica (Bencao Gangmu) gave it a name, that is, “its roots are attached to each other continuously and it can combine incense, so it is called Xiangfuzi.” Recording xiangfu can dissipate qi, cold epidemic, sanjiao, six yu, eliminate dietary accumulation, phlegm and drink ruffian, tarsioma abdominal distension, beriberi, stop pain in the heart and abdomen, ulcer, hematuria and blood under vomiting blood, women with bleeding and leakage, moon syndrome, prefetal and postpartum diseases. “The Book of Materia Medica Tujing” records: “Sedge root, also known as Xiangfuzi... Today shortcut living, seedling leaves such as and thin, root such as Zhu head. February and August.” The Song Dynasty “Bencao Tu Jing” recorded that Xiangfu can be used to treat the heart of the heat, the bladder is connected with hypoxia qi. Those who are sad and unhappy every day, and sleepy heart. “Materia Medica Yan Yi” records: “Sedge, its root such as jujube stone, also called the Xiangfuzi.” The character “Xiang” reflects its aroma, while the character “Fu” reflects that its drug site is the root of the root, rather than the whole root. Li Shizhen in “Compendium of Materia Medica” contained: “BieLu” stop cloud sedge, do not say with seedlings with roots, the name of caryophyte, and do not know the name of sedge also. The grass can be a hat and raincoat, sparse but not stick, so the word from the grass from Sha.” Therefore, *Cyperus rotundus* is called in Jiangsu Province, China.

After textual research, the name of Xiangfu was not unified before the Song Dynasty, and it was gradually standardized from the Song Dynasty to the Ming and Qing Dynasties. Generally, the name of Xiangfu was “Cyperus, Xiangfuzi.” The herbal literature in the late Qing Dynasty was generally collected in the name of Xiangfu. Table 1 shows some of the major traditional clinical applications of CR. Cyperus officinalis can not only be used alone for the treatment of various diseases, but also can be used in combination with other drugs (Wang et al., 2019). Table 2 lists the drug pair application of CR in detail.

**TABLE 1:** Traditional application of CR in several countries or districts.

Country/District	Local name	Traditional application	Reference
China	Xiang fu/Suo chao gen	Mainly used to remove heat in the chest, take a long time can fill the air, promote eyebrows and whiskers growth	Ming Yi Bie Lu (名医别录)
China	Xiang fu/Suo chao gen	Reduce the qi and remove the heat in chest and abdomen	Newly Revise Materia Medica (新修本草)
China	Xiang fu/Suo chao gen	Treat the heart fever, bladder and hypochondriac gas often sad not happy, and the heart	Ben Cao Tu Jing (本草图经)
China	Xiang Fu/Suo chao gen	Cure leaks and drives out clots	Material Medica for Decoction (汤液本草)
China	Xiang fu/Suo chao gen	Disperses the depressive fast qi, disperses the qi cold epidemic disease, the three jiao, solves the six depressive, eliminates the diet accumulation, phlegm drinks the ruffian full, abdominal distension, beriberi, pain in the head and teeth of the stomach and abdomen, sore ulcer, hemorrhea and blood in the urine under vomiting blood, female rupture and leakage, irregular month and month, all kinds of diseases before and after pregnancy	Compendium of Materia Medica (本草纲目)
China	Xiang fu/Suo chao gen	Cure abdominal pain, accumulation of lump, immaculate, leaking, bleeding, alleviating the symptoms and relieving the immobility	Ben Cao Hui Yang (本草汇言)
China	Xiang fu/Suo chao gen	Open the yu shuqi, eliminate the wind itch	Lei Gong Pao Zhi Yao Xing Jie (雷公炮制药性解)
China	Xiang fu/Suo chao gen	Open liuyu, eliminate phlegm food, scattered wind and cold, blood qi, stop all pain, month hou is not adjusted, collapse fetal birth	Ben Cao Tong Xuan (本草通玄)
China	Xiang fu/Suo chao gen	Eliminate phlegm and open depression, relieve anxiety and dispel suspicion, stop hypochchia pain and abdominal distension and pain, treat chest heat and epigastolic distress, commonly used in fetal and childbirth, and is suitable for female departments, twelve channels, and the triple energizer is open	Ben Cao Zheng Yao (本草征要)
China	Xiang fu/Suo chao gen	Cure cold and skin rash, chest deficiency heat, promote digest, promote the triple energizer, remove the six depressions (phlegm depression, fire depression, qi depression, blood depression, dampness depression, food depression), and stop all kinds of pain	Yi Xue Ru Men · materia medica (医学入门·本草)
China	Xiang fu/Suo chao gen	Cure many anger and worry, full phlegm and drink, chest swelling and abdominal distension, diet accumulation, cholera vomiting and diarrhea, kidney gas and beriberi, sore ulcer (caused by blood coagulation and qi stagnation)	Ben Cao Bei Yao (本草备要)
China	Xiang fu/Suo chao gen	Relieve qi depression and qi pain, regulating menstruation and removing blood stasis, removing skin pruritus, stopping cholera vomiting and reflux, hemorrhaging of blood, swelling and carbuncle of breast, all can be used to treat food and stay away, diarrhea can be fixed, long hair, blood leading medicine to qi, this is the product must be used in qi and blood	Ben Cao Xin Bian (本草新编)
China	Xiang fu/Suo chao gen	Enter the pulse, open depressive qi, eliminate phlegm food, scattered cold, blood qi, stop the pain month is not regulated, childbirth leakage, more anger more worry to the drug treatment of two hypochondria qi, slow heart, is the blood of the gas medicine	Ben Jing Feng Yuan (本经逢原)
India	Musta/Nagarmotha/Motha	Fever, dysentery, itching, pain, vomiting and various blood diseases, anti-inflammatory, antipyretic, analgesic, antidiarrheal and antimalarial effects	Badgujar and Bandivdekar (2015)
Japan	Koubushi	Thirst, fever and blood diseases	Ito et al. (2012)
Pakistan	—	Stomach diseases	Arshad et al. (2014)
South Korea	Purple nutsedge/Koubushi	Gynecological and inflammatory diseases	Seo et al. (2014)
Sudan	Cyperaceae	Stomach disorders, bowel irritation, dyspepsia, diarrhoea	El-Ghazali et al. (1994)
Sudan	Ya haeo mu	Flatulence, haemorrhoids, constipation	Oratai et al. (2017)

**TABLE 2:** Compatibility of CR.

Combination	Application
Chuanxiong Rhizoma	The compatibility of the two can strengthen the effect of qi relieving depression, treating chest and diaphragm distress, abdominal distension and pain, mal-decay and swallowing acid, nausea and vomiting, and dietary deprivation, etc
Bupleuri Radix	The compatibility of the two have the effect of soothing liver, regulating qi and relieving pain, and treating liver qi stagnation syndrome
Alpiniae Officinarum Rhizoma	The compatibility of the two have the effect of warming and dispersing cold, regulating qi and relieving pain, treating qi stagnation and cold coagulation syndrome, such as epigastric pain, chest swelling, fear of cold and love heat, women dyspenorrhea, etc
Linderae Radix	The compatibility of the two have the effect of regulate qi and relieve depression, disperse cold and relieve pain, treat chest and diaphragm pain, even flank and umbilicus, and remove undetermined
Atractylodis Rhizoma	The compatibility of the two can treat the shape sheng phlegm, qi deficiency
Toosendan Fructus	The compatibility of the two can relieve pain by relieving qi
Corydalis Rhizoma	The compatibility of the two have the effect of regulating qi, dredging collateral and relieving pain, and treating metrorrhagia and metrostaxis
Aucklandiae Radix	The compatibility of the two can treat pelvic inflammatory disease, qi and pain relief
Angelicae Sinensis Radix	The compatibility of the two have the effect of enriching blood, activating blood and dredging collaterals, and is used to treat intrauterine adhesions
Leonuri Herba	The compatibility of the two have the effect of regulating qi and guiding hysteresis, and is used to treat ulcerative colitis
Leonuri Herba	The compatibility of the two have the effect of regulating qi, activating blood, regulating menstruation and relieving pain. It is used to treat primary dyspenorrhea
Santali Albi Lignum	The compatibility of the two have the effect of activating blood, regulating qi and regulating menstruation, and is used for pelvic inflammatory infertility

### 3.2 Prescription applications

There are hundreds of clinical prescriptions containing CR. Table 3 lists some representative formulations and others are listed in Supplementary Table A2. The formulations involved in the prescription included tablets, capsules, granules, pills, powders, ointments, etc. Among them, Yueju pill, Xiangfu pill, Qizhixiangfu pill, Sizhixiangfu pill are the most widely used in clinic, which have the functions of regulating qi and resolving depression, soothing liver and regulating qi, nourishing blood and regulating menstruation, soothing liver and invigorating spleen. It is commonly used in clinical practice for diseases such as epigastric stuffiness, abdominal distention, food stagnation, belching and acid swallowing, irregular menstruation caused by liver depression and blood deficiency, and spleen dysfunction, pre-and post-menstrual symptoms, blood deficiency and qi stagnation, irregular menstruation, chest and abdominal pain.

**TABLE 3:** Representative clinical prescriptions for CR.

Prescription name	Main components	Formulation	Traditional and clinical uses	Reference
Kuanqi Tang	CR	Decoction	Cure all qi disease heart abdominal distension full, chest diaphragm choking, sour breath swallowing, sputum and vomiting in the stomach and drink, do not think about diet.	Prescription of the Bureau of Taiping People’s Welfare Pharmacy (太平惠民和剂局方)
	Shrinkage amomum Villosum			
	Licorice			
Xiaowuchen Tang	Radix linderae	Decoction	Cure the tingling pain in the heart, regulate the middle and fast qi.	Prescription of the Bureau of Taiping People’s Welfare Pharmacy (太平惠民和剂局方)
	Licorice			
	CR			
Yueju Wan	Rhizoma atractylodis	Pill	Resolve all depression.	Dan Xi Xin Fa (丹溪心法)
	CR			
	Stroke men			
	The divine comedy gardenia			
Xiangjin San	CR	Powder	Treat anal prolapse.	San Ying Ji Yi Bin Zheng Fang Lun (三因极一病证方论)
	Spica Schizonepetae			
Tiezhao San	CR	Powder	Miscarriage prevention.	Zhong Cang Jing (中藏经)
	Thick fried basil soup			
Cufu Wan	CR	Pill	Governing yuan viscera deficiency and cold,dizzy, eat less, cold and hot all over, acute abdominal pain, red leucorrhea, heart palpitation, Qi tightness, deficiency cold in blood and unstable fetal Qi.	Fu Ren Liang Fang (妇人良方)
	Herba leonuri			
	Angelica sinensis			
	Rehmannia glutinosa			
	Radix bupleuri			
	Rhizoma ligustici Wallichii			
Xiangsu San	Pericarpium citri reticulatae	Powder	To treat plague and typhoid fever.	Prescription of the Bureau of Taiping People’s Welfare Pharmacy)太平惠民和剂局方)
	CR			
	Perilla leaf			
	Licorice			
Xiangsu Congchi Tang	CR	Decoction	Pregnancy typhoid fever, aversion to cold and fever, no sweat, head and body pain, stuffy chest cavity, thin white moss, floating pulse	Reformulate the popular theory of febrile diseases (重订通俗伤寒论)
	Will the new skin			
	Fresh worship			
	Perilla			
	Clear the main grass			
Jiawei Xiangsu San	Perilla leaf	Power	Exogenous wind and cold, accompanied by qi stagnation syndrome, headache, nasal congestion and runny nose, body pain, fever, aversion to cold or wind, no sweat, stuffy chest cavity, thin white moss, floating pulse.	Yi Xue Xin Yu (医学心悟)
	Pericarpium citri reticulatae			
	CR			
	Licorice			
Laingxue Qingchang San (Tang)	Rehmannia glutinosa	Power (Decoction)	Harmonizing intestine-stomach.	Zheng Zhi Zhun Sheng (证治准绳)
	Radix paeoniae alba			
	Herba schizonepetae			
	Yellow shall			
	CR			

### 3.3 Other applications

CR is not only used in medicine, but also in aquaculture, health care, food processing and other fields (Supplementary Table A3). In addition, the tubers and root of CR have been used in Arab countries, Africa, China and India for centuries for perfume, spices and Ayurvedic therapy (Sharma and Gupta 2007). The leaves of CR are widely used to season food, especially in the Middle East and Southeast Asia. Its seeds are also raw materials for curry, salted spices, Greek dishes and baking products (Jabier et al., 2008).

## 4 Phytochemistry

Up to now, there are many studies on the chemical constituents of CR. Studies have shown that CR contains a variety of active components, mainly including volatile oil, flavonoids, alkaloids, carbohydrates, saponins, phenols, triterpenoids, anthraquinones, diterpenes and trace elements (Chen et al., 2017). This chapter classifies the chemical constituents isolated and identified by scholars in the form of tables (Supplementary Table A4), and introduces the corresponding structure in the form of figures.

### 4.1 Volatile oil

**FIGURE 2:**
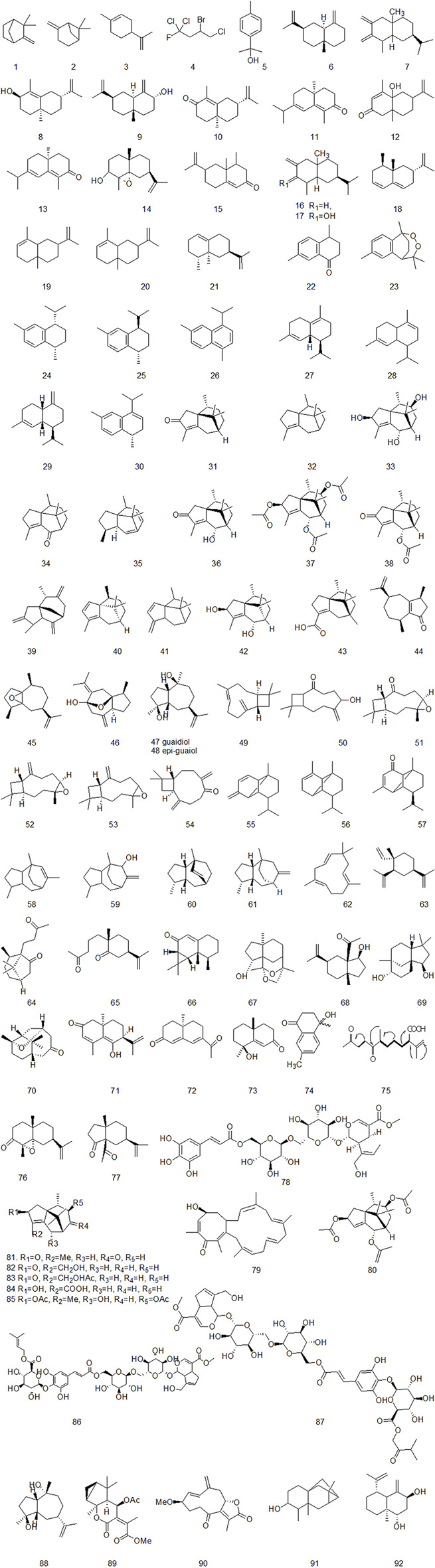
The volatile oil components of CR that have been isolated so far.

The essential oil is the main active ingredient of CR, which is mainly studied by gas chromatography-mass spectrometry (GC-MS) and high-performance liquid chromatography (HPLC) (Aghassi et al., 2013). At present, nearly 100 volatile oil components of CR have been identified. α-Cyperone and Cyperenone are the main components of volatile oil of CR (Guo et al., 2019). Most of the volatile oils of CR are sesquiterpenoids. There are relatively many sesquiterpenes in the identified and isolated volatile oil compounds, including cineolane sesquiterpenes, syringane sesquiterpenes, guaiacane sesquiterpenes, pogostemane sesquiterpenes, gins sesquiterpenes, piperane sesquiterpenes and monocyclic sesquiterpenes, as shown in Figure 2 (Thebtaranonth et al., 1995; Jin et al., 2011; Khan et al., 2011; Ahn et al., 2015; Ryu et al., 2015; Xu et al., 2015).

The volatile oil of CR has the effects of antioxidant, anti-inflammatory, antibacterial, analgesic, estrogen-like, and insecticidal. Ardestani et al. through ABTS, 1,1-diphenyl-2-picrylhydrazyl (DPPH) free radical scavenging capacity test, Ferric ion reducing antioxidant power (FRAP) test, Cu, AAPH + induced protein oxidative damage protection test, Fe, AAPH2 + induced DNA oxidative damage protection test showed that the caryophyllene, α-caryophyllone can play an antioxidant role by scavenging free radicals and oxidative damage to proteins (Ardestani and Yazdanparast 2007). Wu Sha et al. used acetic acid writhing reaction in mice and hot plate test in mice to prove that Cyperenone and α-Cyperone have analgesic effect, but the specific mechanism is still unclear (Fan et al., 2017; Wu et al., 2018). Zhang et al. tested the antibacterial activity by Oxford cup method, MIC and MBC by tube double dilution method, growth curve detection, flow cytometry to detect the effect of volatile oil of CR on bacterial mortality. The results showed that Cyperene I and II had strong antibacterial effect, and may be related to the promotion of protein denaturation in cell membrane and the destruction of membrane permeability by phospholipid reaction (Zhang et al., 2017; Khojaste et al., 2018). Ding et al. (2015) through the contraction experiment of isolated uterine smooth muscle in mice and the oxytocin-induced dysmenorrhea experiment in mice, showed that α-Cyperone might be involved in inhibiting the phosphorylation level of p42/44MAPK and the expression of Cx43, and regulating the balance of TXB2/6-Keto-PGF1α and the related path of antioxidant effect to exert estrogen-like effect. In addition, α-Cyperone can also affect the digestive system of animals by promoting the biological activity of gastrointestinal motility and promoting the proliferation of small intestinal smooth muscle cells (Zhang et al., 2015). Nootkatone can inhibit platelet aggregation induced by collagen, thrombin or maximum platelet aggregation (Seo et al., 2011). The active monomer compounds of essential oil were mainly studied in sesquiterpenes.

### 4.2 Flavonoids

**FIGURE 3:**
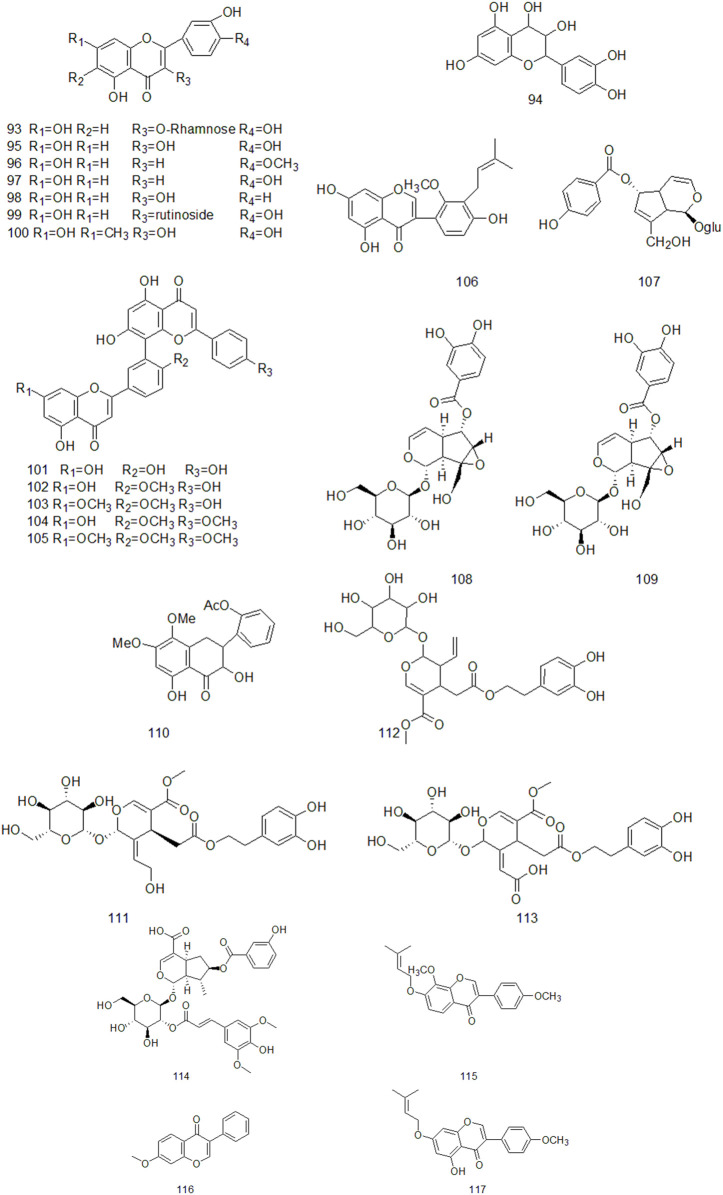
The flavonoids components of CR that have been isolated so far.

Flavonoids are widely found in plants, which are composed of two molecular benzene rings connected by intermediate three carbon atoms, and can be summarized as C6-C3-C6 structures in structure. (Li and Liang, 2021). Flavonoids are secondary metabolites of plants and have high medicinal value (Echeverry et al., 2015). The results of Huang Suoyi et al. ‘s study on the extraction of total flavonoids from CR showed that the content of total flavonoids was 0.1864 mg/ml and the recovery was 102.7%, indicating that the purity and yield of total flavonoids in CR were higher (Huang et al., 2008). Xu Yan et al. used chromatography technology to isolate and purify the ethyl acetate part of CR ethanol extract and obtained nine flavonoids, including four single flavonoids and five double flavonoids, which were identified as: Kaempferol, luteolin, quercetin, Phellodendron flavone, Picotaxa spirotaxa diflavone, demethylginkgo biloba diflavone, Ginkgo biloba diflavone, isoginkgo biloba diflavone, golden pine diflavone (Xu et al., 2010). At present, more than 20 flavonoids have been isolated from CR (Figure 3).

Sunil found that total oligoflavones (TOFs) of CR can reduce glutamate and glutamine synthetase, increase Na + K + atp enzyme activity, and have neuroprotective effect. At the same time, TOFs can significantly reduce the neurological deficits of rats and reverse their anxiety behaviors, reduce the MDA content in brain tissue of rats, and increase the contents of superoxide dismutase and glutathione. TOFs may be a potential candidate for drug development for stroke treatment in the future (Sunil et al., 2011). In addition, flavonoids in CR also have the activities of scavenging DPPH, metal chelating, 2,2′-N-bis (3-ethylbenzothiazolin-6-sulfonic acid), scavenging NO and hydroxyl free radicals, and have antioxidant effects (Kandikattu et al., 2015).

### 4.3 Triterpenes and sterols

Triterpene compounds have diverse structures and various types. According to the structure and properties of compounds, they can be divided into triterpenoid saponins, sterol glycosides and other triterpenoids (Dai et al., 2021). Zhou (2012) proved that triterpenoids of CR have anti-inflammatory effect through acute inflammation model using carrageenan-induced foot swelling in rats, egg white-induced foot swelling in rats, and xylene-induced ear swelling in mice. At the same time, he proved that triterpenoids had antipyretic effect through 2,4 - dinitrophenol-induced fever model in rats.

**FIGURE 4:**
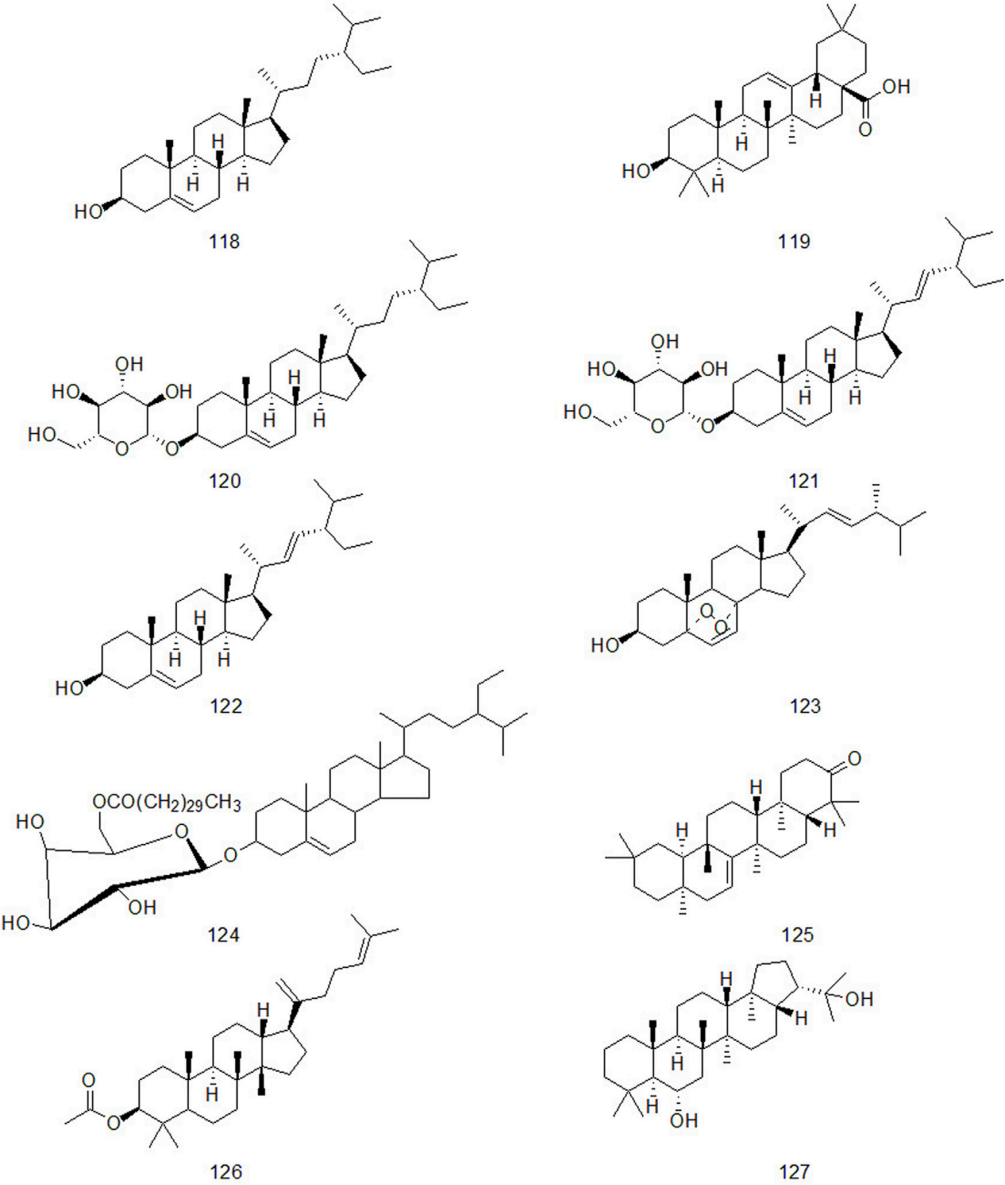
Thet triterpenes and sterols components of CR that have been isolated so far.

CR contains not only triterpenoids, but also sterols. Studies have confirmed that the natural phytosterol β-sitosterol in CR has obvious pharmacological effects such as lowering cholesterol, anti-inflammatory, antitussive and anti-cancer (Piao et al., 2015; Ma et al., 2017; Tianhao et al., 2018; Yang et al., 2020; Chen et al., 2021). The triterpenes and sterols in CR are shown in Figure 4.

### 4.4 Others

**FIGURE 5:**
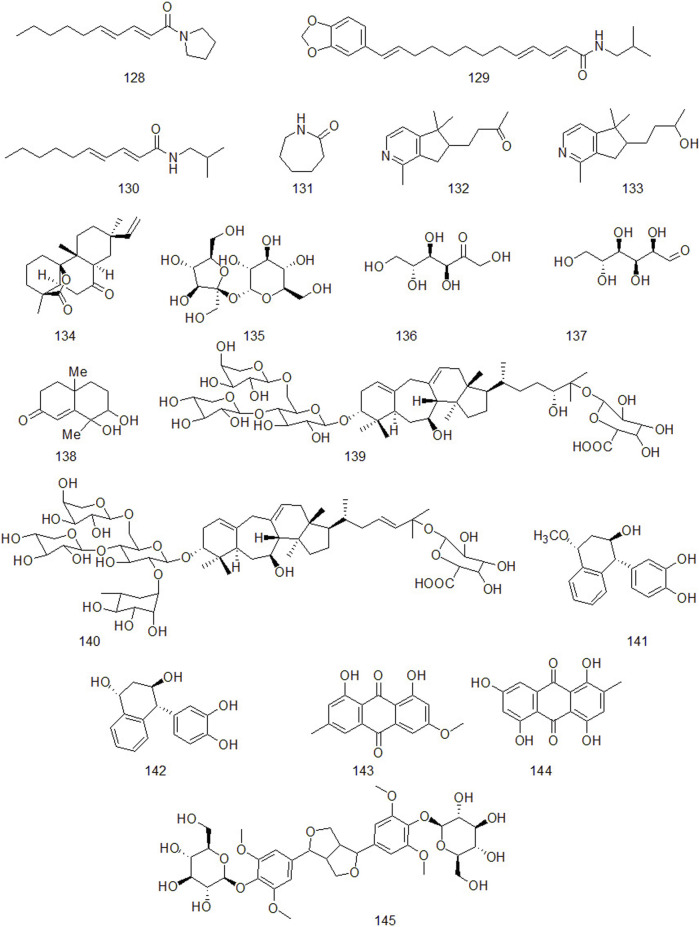
The others components of CR that have been isolated so far.

Natural alkaloids are a class of basic nitrogenous organic compounds with important biological activities. According to their different chemical structures, they can be divided into isoquinoline, pyrrole, pyridine, quinoline and indole, etc (Li and Zeng, 2021). At the same time, alkaloids are widely distributed in plants of Ranunculaceae, Papaveraceae, Menispermaceae, Solanaceae, Rutaceae, Leguminosae and Polygonum cuspidatum, which have anti-tumor, anti-inflammatory, analgesic, antibacterial, antiviral, insecticidal and cardiovascular diseases. Alkaloids contained in CR are shown in Figure 5.

Xu et al. isolated a diterpenoid compound from CR: rosenonolactone (Xu et al., 2008). Wen et al. (2003) extracted the extract with ethanol from CR and separated sucrose compounds in methanol elution. In addition, the carbohydrates isolated from CR: include D-fructose and D-glucose (Asenjo, 2010). Luo et al. (2014) used silica gel column chromatography, gel column chromatography, thin layer preparation and other methods to isolate the chemical constituents of CR and the structure of the compounds was analyzed by means of nuclear magnetic resonance. A compound which had not been reported in literature was isolated from CR and for the first time. It was speculated to be a configurational isomer of Oxyphyllenone and was named oxyphyllenone C. Lin et al. (2017) obtained two new cycloartane glycosides from the part of n-butanol extracted from CR after 95% ethanol extraction: Cyprotuoside C and Cyprotuoside D. Zhou and Yin (2012) isolated two novel phenolic compounds from ethyl acetate fractions of CR:1α-methoxy-3β-hydroxy-4α-(3′,4′-dihydroxyphenyl)-1,2,3,4-tetrahydronaphthalin and 1α,3β-di-hydroxy-4α-(3′,4′-dihydroxyphenyl)-1,2,3,4-tetrahydronaphthalin. CR also contains anthraquinones, such as emodin methyl ether, Catenarin, liriodendrin, etc (Wu 2009; Xu et al., 2016). In 2021, Reham et al. isolated a new cytotoxic ceramide from CR guided by bioanalysis (Samra et al., 2021). In addition, it also contains rich trace elements, such as Fe, P, Ca, Sr, Mn, Zn and Al, etc (Jin et al., 1998).

## 5 Pharmacology

### 5.1 Anti-depression

Depression is a kind of mental illness which is caused by many factors with the main symptoms of continuous low mood and cognitive dysfunction. The clinical manifestations of depression are various, and it has long plagued human psychological and physiological health (Nestler e al., 2002). Depression is increasingly common and serious, and constitutes a major health problem. In 2008, WHO listed severe depression as the third leading cause of the global burden of disease and predicted it to rank first by 2030. In traditional Chinese medicine, depression has been considered as a disorder of qi in the body, and CR is one of the commonly used drugs to treat depression.

Zhou and Liu (2012) used two classical behavioral models for antidepressant screening, namely, the forced swimming test (FST) and the tail suspension test (TST), to evaluate the antidepressant effects of the iridoid glycosides extracted from CR. The results showed that rotunduside G and rotunduside H had significant antidepressant activity. However, this experiment was conducted only at the animal level, without examining the intracellular mechanism and clinical level. Lin et al. (2017) used the FST and open field test (OFT) models to evaluate the antidepressant activity of the extract and its components of CR. The results showed that the extract of CR, Rotundine F, showed significant antidepressant activity at a dose of 50 mg/kg, which was close to the positive control fluoxetine (20 mg/kg). However, there was only one dose in this experiment, and the dose dependence was not investigated. Hao et al. (2017) used FST and TST to study the antidepressant. Effect of CR extract, and found that CR extract significantly inhibited the activity of Monoamine oxidize A (MAO) in the whole brain of rats. The results suggested that the antidepressant effect of CR extract in the rat may be related to MAO inhibitory activity in a dose-dependent manner. But the study was conducted only at the animal level, without investigating the intracellular mechanism and clinical level.

Wang et al. used FST, TST and OFT to observe antidepressant effects of the 95% ethanol extract (XFE) and the water extract (XFW) isolated from CR. The results confirmed for the first time that XFE was the active extract of CR with antidepressant effect, and further clarified that the active extract XFE showed a good dose-effect relationship in the dose range of 0.5–2 g/kg (Wang et al., 2013). However, this experiment did not determine the specific active ingredients and mechanism of antidepressant of CR, so more *in vivo* experiments are needed to determine the possible role and metabolic mechanism of CR on depression. Zhou and Liu (2012) used mouse FST and TST to screen the antidepressant activity of alcohol extract and its polar parts, and then used reversed-phase high performance liquid chromatography to determine the content changes of monoamine neurotransmitter in the frontal cortex of the brain, and to explore the mechanism of antidepressant action of CR.The results showed that the ethyl acetate extract and n-butanol extract of CR had obvious antidepressant effects on the animal model of “behavioral despair”, which might be related to the regulation of the contents of monoamine neurotransmitters 5-HT and DA in the brain. However, in this experiment, the alcohol extract of CR was only investigated, and the physicochemical properties and mechanism of CR, the main antidepressant active substance, were not studied. Jia et al. used the network pharmacology method to analyze the action pathways of the antidepressant chemical component of CR and predict its antidepressant drug effect component and related action targets. The results showed that CR played an antidepressant role by regulating signaling pathways such as adhesion patches, neurotrophic factors, and vascular endothelial growth factor (VEGF) (HongMei et al., 2019). Yanan et al. conducted enrichment analysis, molecular docking, molecular dynamics simulation and molecular mechanics Poisson-Boltzmann surface area by screening active compounds, target prediction, constructing protein-protein interaction network, gene ontology (GO) and Kyoto gene and genome encyclopedia (KEGG) pathway. To investigate the antidepressant mechanism of CR-Chuanxiong Rhizoma herb pair. The results showed that AKT1, IL-6, TP53, DRD2, MAPK1, NR3C1, TNF may be the core target GO enrichment analysis showed that RNA polymerase II promoter, plasma membrane and protein binding were important for the positive regulation of transcription. Neuroactive ligand-receptor interaction, PI3K-Akt signaling pathway, dopaminergic synapse and mTOR signaling pathway are important pathways. Molecular docking results showed that the core compounds and core targets had good binding affinity (Shi et al., 2021). However, although the above studies predicted the antidepressant mechanism of CR, they did not carry out *in vivo* and *in vitro* verification and lacked scientific and reliable data support. Yuhe et al. identified active compounds of CR and potential targets for major depressive disorder (MDD) by network pharmacology. GO and KEGG enrichment analysis showed that the anti-MDD effect of CR may be mediated by changes in liver response to lipopolysaccharide (LPS) and glucose metabolism. Comparing the bioinformatics analysis results of normal and severe diabetic liver tissues of spontaneous diabetic rats, Yuhe et al. selected PAI-1 (SERPINE1) as the CR target for anti-severe diabetes. Molecular docking and molecular dynamics analysis verified the binding of active compound quercetin to PAI-1. The results showed that quercetin was an active compound that enhanced immune response and glucose metabolism by targeting PAI-1. This study not only revealed the material basis and potential mechanism of CR in the treatment of MDD by soothing liver, but also provided evidence for PAI-1 as a potential target and quercetin as a potential target for MDD treatment (Lei et al., 2021).

### 5.2 Hypoglycemic

All types of diabetes are characterized by chronic hyperglycemia. Diabetes is the main cause of blindness, end-stage renal disease and various frail neurological diseases. Diabetes is also associated with accelerated atherosclerotic macrovascular disease, affecting the supply of arteries to the heart, brain and lower limbs (Chithran et al., 2012). Ardestani et al. used fructose-mediated protein glycosylation model *in vitro* to study the hypoglycemic effect of ethanol extract from CR. The results showed that fructose not only enhanced the fluorescence intensity of glycosylated bovine serum albumin (BSA), but also caused more protein hydroxylation and sulfur oxidation after 14 days of exposure to AGEs. By observing the fluorescence intensity of BSA, different concentrations of ethanol extract of CR (25–250 mg/ml) can significantly inhibit the formation of AGEs and protein oxidation, indicating that ethanol extract of CR has a good hypoglycemic effect (Ardestani and Yazdanparast, 2007). However, this study only investigated the aqueous ethanol extract of CR, and no specific active substances were found. At the same time, this study was only verified at the cellular level, and there was no further evidence *in vivo*. Nishikant et al. used alloxan-induced diabetic rat model to study the effect of alcohol extract of CR on blood glucose in rats after oral administration. The administration dose was 200, 500 mg/kg once a day for seven consecutive days. The results showed that the high-dose group (500 mg/kg) significantly decreased the blood glucose level of rats, and there was a significant difference compared with the positive control (metformin) (*p* < 0.05). It was considered that its hypoglycemic effect might be related to the antioxidant effect of CR (Raut and Gaikwad, 2006). However, the ethanol extract of CR studied in this experiment is a mixed component. Whether its effect on blood glucose reduction is the result of a single component or the combined effect of multiple compounds still needs further exploration. Singh P et al. used the diabetes model induced by streptozotocin (STZ) in Swiss mice to study the anti-diabetic activity of ethanol extract of CR (EECR). After 3 weeks of EECR, the body weight, blood glucose, biomarkers (SGPT, SGOT) and blood lipid levels of STZ-induced diabetic mice were measured. EECR showed significant anti-diabetic activity, improved body weight and reduced elevated biochemical parameters, such as SGPT, SGOT, cholesterol and triglyceride levels. The experimental results showed that EECR had obvious anti-hyperglycemic activity in STZ-induced diabetic mice (Singh et al., 2015). However, this study did not determine the specific active components and mechanism of hypoglycemic effect of CR, so more experimental studies were needed to determine the mechanism of hypoglycemic effect of CR.

Almahi I. Mohamed et al. studied the antioxidant and anti-diabetic effects of CR *in vitro* and silicon models. FRAP and DPPH were used to screen the antioxidant activity of crude extracts (ethyl acetate, ethanol and water) *in vitro*, and the inhibitory effect on a-glucosidase. The possible bioactive components were identified by GC-MS. The molecular docking of the selected phenolic compounds was calculated to determine the inhibition mode of a-glucosidase activity. The total phenolic content of water extract was the highest, and the scavenging activity was significantly higher than that of ethyl acetate and ethanol extract. In FRAP and DPPH methods, the IC50 values of the aqueous extract were 448.626 and 418.74 mg/ml, respectively. The inhibitory effect of aqueous extract on a-glucosidase activity was higher, with IC_50_ value of 383.75 mg/ml. GC-MS analysis showed that 4-methyl-2-(2,4,4-trimethylpent-2-yl)phenol, 2-methyl-4-(1,1,3,3-tetramethylbutyl)-phenol and 1-ethoxy-2-isopropylbenzene existed. Molecular docking studies showed that 1-ethoxy-2-isopropylbenzene formed two hydrogen bonds with the interaction residues at the active site of a-glucosidase. The binding energy of 4-methyl-2-(2,4,4-trimethylpentane-2-yl) phenol was the lowest, and its affinity with the active site of glucosidase was the best. These results suggest that CR may have antioxidant and anti-diabetic effects (Mohamed et al., 2021). Luo et al. was used to study the effects of TOF fraction of CR on blood glucose, blood lipid and antioxidant activity of diabetic rats by using a diabetic rat model with high fat and high sugar diet and intraperitoneal injection of streptozotocin. After 1 month of administration, the blood, liver and kidney of rats in each group were taken, and the blood glucose level was detected. The contents of triglyceride (TG), total cholesterol (TC), low density lipoprotein cholesterol (LDL-C) and high density lipoprotein cholesterol (HDL-C) in serum were detected. The contents of catalase (CAT), glutathione peroxidase (GSH-PX) and superoxide dismutase (SOD) in serum, liver and kidney homogenate were detected. After administration, the levels of blood glucose, TG, TC and LDL-C in TOF fraction of CR high, medium and low dose groups were significantly lower than those in model group (10 mg/kg metformin), and the HDL-C value was significantly higher than that in model group (*p* < 0.05). After administration, the contents of CAT, SOD and GSH-PX in serum, kidney and liver homogenate in TOF fraction of CR high, medium and low dose groups were significantly higher than those in model group (*p* < 0.05). The results showed that TOF fraction of CR had a good therapeutic effect on diabetic rats, and it could effectively reduce blood glucose, regulate blood lipid and oxidative stress disorder (Luo and Huang, 2017). Although the high, medium and low doses of TOF fraction of CR were investigated in this study, the optimal effective dose was not determined. At the same time, this study only carried out experiments at the animal level, without investigating the intracellular mechanism and clinical level. The CR-loaded ZnO nanoparticles synthesized by Liwei et al. have also been shown to have better antidiabetic properties (Zhang et al., 2020).

### 5.3 Anti-oxidation

Free radicals are intermediate metabolites of various biochemical reactions in human life activities, with high activity. When the body‘s antioxidant defense capacity is reduced, the balance between free radical generation and elimination is broken, and oxidative stress occurs. Oxygen can produce a variety of active free radicals in the process of metabolism. Polyunsaturated fatty acids in biofilms can undergo lipid peroxidation through these free radicals. They can also cause cell damage through the decomposition products of hydrogen peroxide, thereby affecting cell function, leading to infection, cancer, cardiovascular disease and aging (Li and Zhan, 2018). Xiao et al. (2012) determined the scavenging effect and reducing power of flavonoids from CR on DPPH, OH and O^2-^ by colorimetric method. Flavonoids from CR had obvious scavenging effects on the three free radicals, and the order of scavenging capacity was DPPH>OH>O^2−^. There was a significant dose-effect relationship between the scavenging rate and concentration. When the concentration of flavonoids was 0.4 mg/L, the scavenging rate of O^2−^, DPPH· and ·OH was 25.5%, 67.0 % and 28.5%, respectively. The ability of reducing Fe^3+^ increased with the concentration of flavonoids. Thus, flavonoids from CR have good antioxidant activity. However, this study did not select the positive control as a reference for the antioxidant of flavonoids from CR and lacked the support of experimental and clinical data *in vivo*. Luo et al. intervened with high, medium and low doses of total flavonoids from CR and determined the contents of catalase, superoxide dismutase and glutathione peroxidase. After administration, compared with the model group, the contents of CAT, SOD and GSH-PX in serum, kidney homogenate and liver homogenate of the blank group, positive drug group, high dose group, middle dose group and low dose group were significantly increased, and the differences were statistically significant (*p* < 0.05). The results showed that the expression of the above three antioxidant factors were increased, indicating that total flavonoids from CR have good anti-oxidative stress effect (Luo and Huang, 2017). But the study was conducted only at the animal level, without investigating the intracellular mechanism and clinical level, and without determining the optimal amount of antioxidants. Kandikattu et al. (2015) determined the antioxidant capacity of TOF of CR by measuring the scavenging capacity of TOF on DPPH, metal chelating, ABTS, NO and hydroxyl free radicals. TOF had the activities of scavenging DPPH, metal chelating, ABTS, NO and hydroxyl free radicals, which indicated that TOF of CR have good antioxidant activity. However, this experiment only studied the antioxidant and free radical scavenging activities of total oligomeric flavonoid fraction of CR *in vitro*, lacking intracellular and *in vivo* studies. Mohamed et al. (2021) also proved that Xiangfu has antioxidant effect. Kilani et al. (2005) used the method of free radical scavenging test to investigate the antioxidant capacity of the extracts from the rhizomes of CR, namely, TOF of CR, ethyl acetate and methanol. The results showed that TOF, ethyl acetate and methanol extracts had obvious scavenging activity on DPPH free radical. However, this experiment lacks the comparison of related active substances and positive drugs, and lacks the study of antioxidant mechanism *in vivo*. Although the free radical scavenging capacities of TOF, ethyl acetate and methanol extracts of CR were investigated in this study, the optimal antioxidant amount was not determined. Hao et al. determined the antioxidant capacity of volatile oil from CR by measuring the scavenging ability of ABTS and DPPH free radicals, the iron reduction ability, and the protective ability against oxidative damage of DNA and protein. The results showed that volatile oil from CR could effectively scavenge free radicals, have excellent iron reduction ability, and can strongly protect plasmid DNA from oxidation by oxidant. Thus, the volatile oil of CR has very good oxidation resistance (Hao). However, in this experiment, the antioxidant activities of volatile oil from CR in different antioxidant systems were still significantly different. The specific reasons for these differences were not given, and further research and demonstration were still needed.

### 5.4 Anti-inflammatory

Inflammation is the first response of the immune system to infection, injury or stimulation. Long-term, dysfunctional and adaptive inflammation is related to many human diseases, including allergy, atherosclerosis, gastroenteritis, arthritis, septic shock and autoimmune diseases. Since the existing synthetic drugs have a number of side effects, it is still necessary to find new drugs, while traditional Chinese medicine has been chosen by most people with relatively small side effects, relatively high drug compliance and relatively affordable price of common drugs. Therefore, as a traditional Chinese medicine with anti-inflammatory effect, CR can be studied in depth.

Xie et al. used the inflammatory models of carrageenin-induced rat foot swelling, egg white-induced rat foot swelling, and xylene-induced mouse auricle swelling to observe the anti-inflammatory effect of volatile oil from CR. The results showed that volatile oil from CR (0.018 ml/kg, 1.0009 ml/kg) had a certain degree of inhibitory effect on rat foot swelling induced by carrageenan and egg white, while volatile oil from CR 0.046 ml/kg, 0.024 ml/kg) had no significant effect on xylene-induced ear swelling in mice, indicating that CR had an inhibitory effect on inflammation (Xie et al., 2005). However, the experiment did not set up the positive control group and the blank group. In addition, the experiment examined the dose dependence, which made the conclusion of the study questioned. Chithran et al. (2012) used carrageenan-induced rat paw swelling test model to evaluate the anti-inflammatory activity of the extracts from CR tubers. Compared with the positive control group of indomethacin, the results showed that ethanol extract of CR tuber had obvious anti-inflammatory effect on carrageenan-induced foot swelling in rats. Ding et al. (2015) used carrageenan induced paw swelling in mice, xylene induced ear swelling in mice, and acetic acid induced capillary permeability increase in mice to investigate the anti-inflammatory effect of Cyperi oil dripping pill. The high dose group of Cyperi oil dripping pill can significantly reduce the swelling of the feet and auricle of mice, and reduce the permeability of the peritoneal capillary in mice, indicating that Cyperi oil dripping pill have obvious anti-inflammatory effect, which may be mainly based on α-cyperone. However, the specific mechanism of anti-inflammatory effect was not investigated in this experiment, and the experiment was only carried out at the animal level, without investigating the intracellular mechanism and clinical level. Rocha et al. (2020) used arachidonic acid and 12-O-tetradecanoylphorbol-13-acetate-induced mouse skin inflammation to evaluate the local anti-inflammatory effect of ethanol extract from CR. The results showed that local application of the extract could reduce ear edema and cell infiltration in acute and chronic skin inflammation models, indicating that the extract may be a potential new treatment option for inflammatory skin diseases. However, the experiment was only carried out at the animal level, and the intracellular mechanism and clinical level were not investigated.

Zhang et al. (2013) established an *in vitro* inflammatory model of chicken alveolar type ii epithelial cells infected with APEC-O78, and studied the effects of different concentrations of perfanone on the secretion of TNF-α and TL-10 and the expression levels of TLR-4 and TLR-5 mRNA. The results showed that cyperone could inhibit the production of proinflammatory factors and increase the production of anti-inflammatory factors in APEC-O78-infected chicken alveolar type Ⅱ epithelial cells through TLR-4 receptor signaling pathway, and then exert its anti-inflammatory effect. However, whether it has an effect on NF-κB and MAPK pathways needs to be further studied. Seo et al. (2016) studied the anti-inflammatory activity of the extract isocyperol in LPS-induced RAW 264.7 cells and determined its molecular mechanism. It was found that isocyperol significantly inhibited the production of nitric oxide (NO) and prostaglandin E2 (PGE2) induced by LPS, and inhibited the expression of inducible nitric oxide synthase (iNOS) and cyclooxygenase-2 induced by LPS at mRNA and protein levels. In addition, isocyperol also down-regulated LPS-induced expression of several proinflammatory cytokines, such as IL-1β, IL-6 and McP-1. Isocyperol treatment inhibited LPS-induced nuclear translocation of macrophages and transcriptional activation of NF-κB. In addition, isocyperol inhibited the activation of STAT3, another proinflammatory signal, in LPS-stimulated RAW 264.7 cells. Isocyperol pretreatment can also induce the expression of heme oxygenase-1 (HO-1) in macrophages and reduce the accumulation of reactive oxygen species (ROS) stimulated by LPS. Isocyperol can significantly improve the survival rate of mice with LPS-induced septic shock, and reduce the serum levels of NO, PGE2 and IL-6. These data suggest that isocyperol inhibits septic shock by inhibiting NF-κB, STAT3 pathway and ROS negatively modulating proinflammatory factors. The results showed that isocyperol extracted from Radix aconitum had anti-inflammatory effects. However, the lack of validation in animal and clinical trials, the use of only a single dose, and the lack of dose-dependent studies hinder its wide application in clinical practice.

Tsoyi et al. (2011) used RAW264.7 cells *in vitro* to study the effects of rhizome extract (ECR) on HO-1 induction and inhibition of iNOS/NO production, and whether the anti-inflammatory effects of ECR and its components were related to HO-1 induction. The results showed that ECR could significantly inhibit the expression of iNOS/NO and high mobility group box-1 in macrophages induced by LPS, which was mediated by HO-1 induction. To the attached root extract ingredients screening found such as nookkatone sesquiterpenoids and valencene induced HO-1, this leads to the LPS activated macrophages in iNOS/NO significant inhibition, suggesting that these components associated with the anti-inflammatory properties of ECR. The possible anti-inflammatory mechanism of ECR is due to the induction of HO-1, in which sesquiterpenes such as Nookkatone and Valencene play a key role. However, this study did not examine dose-dependence, nor did it investigate the optimal anti-inflammatory dose. Zou et al. (2017) took the rat model of pelvic inflammatory disease (PID) and the THP-1 cell line stimulated by LPS as the research object, and studied the anti-inflammatory effects of CR, such as inhibiting the infiltration of lymphocytes and neutrophils in the fallopian tube. Reducing the release of interleukin-1β, IL-6, IL-8 and monocyte chemoattractant protein MCP-1 and promote the production of adipoxin A4 (LXA4). The morphological changes of fallopian tube and the levels of inflammatory factors in PID rats were observed. The levels of NF-κB and cytoplasmic NF-κB, IκB-α and FPR2 in THP-1 cells were detected by Western blot. The results showed that CR had anti-inflammatory effects, and its main components were flavonoids, phenols, saponins and alkaloids. The mechanism of its action may be related to the inhibition of NF-κB signaling pathway and the promotion of inflammation resolution. However, this experiment only investigated the crude extract and lacked the comparative study of specific active substances.

### 5.5 Antipyretic and analgesic

Chen et al. (2011) used acetic acid writhing method to investigate the analgesic activity of volatile oil from CR. It was found that the intragastric administration of volatile oil from CR could significantly reduce the writhing times of mice induced by acetic acid, while the non-volatile oil parts of CR had no significant effect, indicating that the volatile oil from CR had obvious analgesic activity. However, there was only one dose of volatile oil in this experiment, and the dose dependence was not investigated. Ou et al. (2004) used aspirin as the positive control, endotoxin-induced fever in rats as the fever model, and physical (hot plate method) and chemical (acetic acid writhing method) stimulation in mice as the analgesia model to study the antipyretic and analgesic effects of different solvent extracts of CR. The results showed that the ethanol extract of CR had obvious antipyretic effect and strong analgesic effect. The aqueous extract of CR also showed strong analgesic effect, but no obvious antipyretic effect was observed. But this study did not study the best safety dose of CR antipyretic analgesic effect, and did not explain the material basis of CR antipyretic analgesic effect. Pal et al. (2009) studied the acetic acid-induced writhing model in mice and observed the acetic acid-induced writhing test in mice. It was found that the acetic acid-induced writhing times in mice were significantly reduced by 1.2% acetic acid, indicating that the ethanol EECR had obvious analgesic effect. Studies have found that the increase in the levels of brain serotonin and GABA can produce analgesic effects, and EECR increases the levels of brain serotonin and GABA in mice. Therefore, the analgesic and anticonvulsant activities produced by EECR may be related to the increase of serum hormone and GABA levels in mice brain. However, this experiment only investigated EECR, lacking the investigation of specific active substances. At the same time, the experiment was only conducted at the animal level, without investigating the intracellular mechanism and clinical level. Xia et al. observes the effect of the extraction parts separated by the organic solvent system of CR on the dysmenorrhea model of mice induced by oxytocin. It is found that the petroleum ether and ethyl acetate parts of CR can significantly reduce the writhing times of mice induced by oxytocin. The petroleum ether, ethyl acetate and medicinal control groups of CR can significantly alleviate the intense contraction of mice uterus induced by oxytocin. Compared with the model group, the petroleum ether group has extremely significant difference (*p* < 0.001), and the ethyl acetate group and the medicinal control group have significant difference (*p* < 0.05). It is confirmed that the petroleum ether and ethyl acetate parts of CR are effective parts for the treatment of dysmenorrhea (Xia et al., 2006). However, this experiment did not determine the specific active ingredients and mechanism of action of CR analgesia, so more experimental studies are needed to determine the mechanism of CR analgesia.

Hussain M et al. studied the analgesic activity of albinism mice by tail-flicking method. The tail of albinism mice was vertically immersed in water at 60°C, and the reaction time was observed after 10 min. The results showed that the deflection time of mice before administration was 3.15–1.11 s, and the deflection time after intragastric administration of CR and aspirin administration were 9.15–0.7 and 9.95–1.11 s, respectively. The tail deflection time of the crude extract of CR was similar to that of aspirin (*p* < 0.05), indicating that its analgesic effect was remarkable (Hussain et al., 2018). This study speculated the possible antipyretic and analgesic mechanism of CR, but this study did not study the best safe dose of antipyretic and analgesic of CR antipyretic analgesic effect, and did not explain the material basis of antipyretic and analgesic of CR effect. Hussain et al. studied the antipyretic ability of crude extract of CR by intraperitoneal injection of brewer’s yeast to induce fever in rabbits (Sun et al., 2003). The results showed that the crude extract of CR could reduce the rectal temperature of rabbits from 40.73 ± 0.98°C to 38.35 ± 0.76°C after 4 h of fever induction, which was significantly lower than that of aspirin from 40.69 ± 0.94°C to 37.90 ± 0.76°C, which indicated that CR had significant antipyretic effect. Flavonoids and alkaloids are known inhibitors of prostaglandins by inhibiting cyclooxygenase, which may be the reason for the significant antipyretic effect (fever) exhibited by the flavonoids of CR. Pain is produced by the interaction of prostaglandins with nociceptors; however, prostaglandins are biosynthesized through the action of cyclooxygenase 1 and 2 on ω-3 and ω-6 polyunsaturated C-20 fatty acids, and the analgesic effect of cyclooxygenase may be mediated by the inhibition of cyclooxygenase, resulting in the failure of prostaglandins to act on nociceptors. Although this study speculated the possible mechanism of antipyretic and analgesic effect of Xiangfu, this study did not study the best safe dose of antipyretic and analgesic effect of CR, and did not explain the material basis of analgesic effect of it. Deng et al. was subjected to structural analysis by ethanol extraction, silica gel column chromatography and nuclear magnetic resonance, and the effective component with antipyretic and analgesic effects in CR was determined as α-Cyperone. The efficacy of α-Cyperone was investigated by endotoxin-induced fever in rabbits, acetic acid writhing test in mice and hot plate test. Compared with the model group, α-Cyperone could reduce the body temperature of endotoxin-induced fever in rabbits (*p* < 0.05). The results showed that α-Cyperone is one of the effective components of CR, and α-Cyperone may play an analgesic effect through peripheral mechanism (Deng et al., 2012). This study showed that the material basis of the antipyretic and analgesic effect of CR was α-cyperone, but the dose-dependence, minimum effective dose and optimal safe dose of the antipyretic and analgesic effect of CR were not investigated. Cheng et al. explored the potential active molecules and corresponding targets of CR in the treatment of primary dysmenorrhea by systematic pharmacology research platform, and screened the therapeutic targets related to primary dysmenorrhea by Uniprot and DAVID databases, and mapped them to KEGG database to analyze its potential mechanism. The results showed that most of the target proteins were concentrated in the VEGF signaling pathway, HIF-1 signaling pathway and Toll-like receptor signaling pathway, and the multiple pathways mapped by the target proteins were mainly related to the regulation of inflammatory factors, prostaglandin synthesis and angiogenesis in patients with primary dysmenorrhea, indicating that CR was treated with multi-molecule, multi-target and multi-pathway for primary dysmenorrhea (Cheng et al., 2019). However, this study only predicted the treatment of primary dysmenorrhea by CR without *in vitro* and *in vivo* verification, and lacked scientific and reliable data to support clinical application.

### 5.6 Anti-tumor

Soumaya et al. was used to detect DNA fragmentation in L1210 cells by 1.5% agarose gel electrophoresis. When the concentration of volatile oil extract of CR was 50 mg/ml, it was clear that DNA was broken, while the control group did not occur. The total flavonoids and ethyl acetate extract of CR can inhibit the reduction of tetrazolium nitroblue by superoxide free radicals in the non-enzymatic superoxide production system, and inhibit the growth and proliferation of mouse lymphocyte L1210 cells, suggesting that CR may have chemotherapy and cell inhibitory activity in mouse leukemia lymphoma (Kilani et al., 2008). P et al. used n-hexane, chloroform, ethyl acetate, methanol and water as solvents to determine its ability to scavenge free radicals by DPPH method. Based on the high antioxidant activity of methanol extract of CR (MRCr), the cytotoxicity of MRCr on human breast cancer (MCF-7), cervical cancer (HeLa), liver cancer (heg2) and prostate cancer (PC-3) was studied. The results showed that MRCr had obvious inhibitory effect and certain protective effect on non-cancer cells (Mannarreddy et al., 2017). However, this study only investigated the crude extract, lacking the comparison of specific active substances. At the same time, this study was only verified at the cell level, lacking further proof of animal *in vivo* studies. Fang et al. (2015) used gastric cancer SGC-7901 cell line as the model to study the anti-tumor effect of extracts from CR. The results showed that the extracts of CR could significantly inhibit the proliferation of gastric cancer cells, and had a dose-effect relationship. With the increase of dose, the anti-tumor effect was enhanced. It was concluded that petroleum ether and chloroform extracts contained key chemicals that inhibited the proliferation of gastric cancer cells, which provided an important reference for the further study of effective active ingredients in CR. However, the extract of CR studied in this experiment is a mixed component. Whether its inhibitory effect on tumor cells is the result of a single component or the combined effect of multiple compounds still needs further exploration.

Song et al. used MTT method and LDH activity determination to determine the dose-effect and time-effect relationship of attar extracted from CR (ACA) extracted by supercritical CO^2^ on killing A549 cells. AnnexinV-EGFP/PI double fluorescence staining was used to observe the apoptosis induced by supercritical CO^2^ extraction of CR oil. The intracellular Ca^2+^, MDA, ATP concentration and SOD activity were determined. Rhodamine 123 was used to detect mitochondrial membrane potential (Δψ) to analyze its mechanism of inducing apoptosis. The results showed that compared with the positive control drug cisplatin, ACA induced apoptosis of lung cancer cells A549 with strong efficacy and rapid onset. ACA induces calcium overload, increases oxidative damage, inhibits energy metabolism and damages mitochondrial structure and function, and finally activates mitochondrial apoptosis pathway to induce apoptosis of A549 cells. Studies have shown that ACA can strongly induce apoptosis to produce anti-lung cancer effect by activating endogenous apoptotic pathways (Song, 2019). But this study only in the cell level, not in the animal and clinical level to verify, only to investigate the crude extract, the lack of specific active substances did not find the contrast, did not explain the material basis of the anti-tumor effect of volatile oil of CR. Ju et al. established the pathological model of uterine leiomyoma by injecting diethylstilbestrol and progesterone into female SD rats. Then the levels of estradiol and progesterone in serum and NOS in uterine leiomyoma tissue homogenate were measured by radioimmunoassay. Apoptosis-promoting genes such as Bcl-2 and Bax were detected by immunohistochemistry to study the anti-hysteromyoma effect of amentoflavone isolated from CR. The results showed that amentoflavone had a significant inhibitory effect on uterine tumors in rats. The mechanism may be by up-regulating the expression of Bax protein and down-regulating the expression of Bcl-2, forming a homodimer Bax/Bax, reducing plasma estradiol and progesterone, and promoting apoptosis of uterine fibroids (Ying and Bing 2016). Although this study elucidated and predicted the mechanism of amentoflavone inhibiting uterine tumor in rats, the maximum safe dose of amentoflavone was not studied. Research on safe dose of clinical medication is also relatively lacking.

### 5.7 Antibacterial

Yazdanian et al. (2018) compared the inhibitory effect of ethanol extract, water extract and volatile oil of CR on Streptococcus mutans, actinomycetes and Candida albicans, and these bacteria are the risk factors of periodontitis and tooth decay. The statistical analysis of the bacteriostatic circle results showed that ethanol extract, water extract and volatile oil of CR had significant inhibitory effect on the growth of Streptococcus mutans and actinomycetes, and ethanol extract had the strongest inhibitory effect, suggesting that CR extract can prevent and treat periodontitis and tooth decay. However, this study only examined the antibacterial effect of extracts, and did not analyze the specific antibacterial active components in CR. Yu et al. (2007) found that the methanol extract of CR powder had a certain preventive effect on dental caries caused by Streptococcus mutans. The inhibitory effect of CR extract on Streptococcus mutans was dose-dependent, and 4 mg/ml extract could significantly reduce the adhesion of Streptococcus to hydroxyapatite coated with saliva. However, this study only studied the antibacterial activity of CR at the cellular level, lacking the comparative study of related active compounds and positive drugs, and lacking the support of animal *in vivo* and clinical experimental data.

Soumaya Kilani et al. test the antibacterial activity of different extracts of CR, namely the freezed infusion, ethyl acetate, methanol and TOF enriched extracts of CR against Salmonella enteritidis, *Staphylococcus aureus* and *Enterococcus faecalis*. The TOF extract of CR had the strongest inhibitory activity against *Staphylococcus aureus* and Salmonella enteritidis, and the inhibitory activity against *Salmonella typhimurium* was obvious. The minimum inhibitory concentration against Salmonella enteritidis was 2.5 mg/ml, and the minimum inhibitory concentration against *Escherichia coli* and *Salmonella typhimurium* was 5 mg/ml or more than 5 mg/ml. The results showed that CR had good antibacterial activity, which could be attributed to polyphenols, tannins, and coumarins found in crude extracts (Kilani et al., 2008). However, this study did not clarify the specific mechanism of its antibacterial effect, so it is necessary to further study its activity *in vivo*, so as to elaborate and develop the prospect of antibacterial. Hao found that CR essential oil had good inhibitory effects on six food spoilage bacteria (*Staphylococcus aureus*, Staphylococcus albus, Bacillus subtilis, *Salmonella typhi*, *Escherichia coli* and Shigella). The results showed that essential oil of CR caused the leakage of intracellular macromolecules from cell membrane lysis, further affected the synthesis of intracellular proteins and DNA, increased the number of apoptosis, and eventually led to cell death, which played an inhibitory role (Hao). However, this study did not explain how the essential oil of CR destroyed the cell wall membrane system of bacteria, and how it affected the synthesis of proteins and DNA, which still needed further study. At the same time, this study only studied the essential oil of CR, lacking the comparison between related active substances and positive drugs, and lacking the study on the mechanism of antibacterial effect *in vivo*.

In addition, studies have shown that, the volatile oil of CR has inhibitory effect on *Staphylococcus aureus*, and has no effect on other bacteria. Cyperene I and II had stronger antibacterial activity than volatile oil, and were also effective for Shigella sonnei. Hydrogenation did not affect the antibacterial effect of Cyperene I and II, while Cyperone was completely ineffective. The extracts of CR also inhibit some fungi (Liu et al., 2009).

### 5.8 Others

In addition to the above effects, CR has also been confirmed to regulate uterine smooth muscle, anti-allergic effects. Li et al. (2007) studied the effect of CR decoction and alcohol precipitation on the isolated uterus of unpregnant rats. The results showed that CR decoction and alcohol precipitation could weaken the contraction movement of isolated uterus smooth muscle of unpregnant rats, slow down the frequency of contraction wave, reduce the amplitude and shorten the duration. It was proposed that the mechanism might be through the synthesis and release of prostaglandin, which was independent of L-type calcium channel, H_1_ receptor and α receptor. Ji-Hye et al. proved in 2022 that CR can inhibit the adhesion of human endometriosis 12Z cells to peritoneal mesothelial Met5A cells. CR extract could down-regulate the mRNA expression of P-cadherin and matrix metalloproteinase-2. In addition, CR significantly inhibited the mRNA expression of neurotrophic factors (BDNF, NGF, NT-3 and NT-4/5) in 12Z cells. At the same time, Akt overexpression significantly neutralized the inhibitory effect of neurotrophic factor expression on cell adhesion in CRE and 12Z cells. Ji-Hye et al. found that aconite inhibited NF-kB activation via Akt pathway. Therefore, CR may exert anti-endometriosis activity by inhibiting the a dhesion of endometriosis cells and the expression of neurotrophic factors and negatively regulating Akt and NF-kB pathways in endometriosis cells (Ahn et al., 2022).

Bae et al. (2010) obtained spleen cells from BALB/c mice, and quantified the secretion of IFN-γ or IL-4 by cell counting. Meanwhile, the secretion of IFN-γ or IL-4 was analyzed by ELISA. The Th1/Th2 polarization experiment showed that the aqueous extract of CR could enhance the secretion of γ-interferon in Th1 cells and reduce the effect of IL-4 in Th2 cells in a dose-dependent manner. Studies have shown that the water extraction of CR can selectively promote the growth of Th cells, and IL-4 in Th2 cells induced B cell antibodies to IgE induced allergic reactions. Therefore, the aqueous extract of CR inhibited the allergic reaction by inhibiting interleukin-4 in Th2 cells. In addition, oral administration of ethanol extract from CR, nootkatone and valenene have significant anti-allergic effects on rats with delayed allergic reaction (Jin et al., 2011). Seo et al. (2011) valuated its antiplatelet activity by observing the effects of ethanolic extract of CR and its eight compounds on platelet aggregation in rats *in vitro* and *in vivo* and the number of tail bleeding in mice. It was found that ethanol extract and monomer compound nootkatone could effectively inhibit platelet aggregation induced by collagen, thrombin or arachidonic acid, and significantly prolong tail bleeding time in mice. The combination of Alpinia officinarum (Zingiberaceae) and CR can also resist gastric ulcer (Qu et al., 2021).In addition to the above effects, CR has also been proved to have estrogenic effects, promoting the decomposition of adipose tissue *in vitro*, lowering blood pressure, anti-ulcer, promoting permeability, anti-mutagenic activity, enhancing immunity and regulating weight (Liu et al., 2009; Cao et al., 2010; Dong-Bao et al., 2017; Kanagali et al., 2022).

At present, the pharmacological activities of the extract of CR are studied in depth. However, the researches on the active material basis of CR are not detailed enough, and the researches on its physical and chemical properties are not deep enough. The mechanism of action in animals is not comprehensive enough. There are also few studies on clinical effective dose and safe dose. The possible reason is that the chemical composition of CR is complex, the pharmacological effects are diverse, the mechanism, target and pathway of action are complex, and it is impossible to conduct a comprehensive and in-depth study of its pharmacological effects and mechanisms in a limited time. It needs the joint efforts of future scholars to clarify the mechanism of different pharmacological effects. The purpose of this part is to summarize the modern pharmacology of CR and analyze the shortcomings of its research, so as to provide reference and guidance for future scholars to study CR.

## 6 Toxicity

Generally speaking, toxicity test of plant drugs is necessary for the safety and reliability of clinical application. In the acute toxicity study of mice, oral ethanol extract of CR 5000 mg/kg b.w did not produce any toxic symptoms, visceral gross appearance changes and behavioral changes or signs of death. In subacute poisoning, repeated oral administration of 1,000 mg/kg b.w ethanol extract of CR for more than 14 days showed that the extract did not cause changes in general behavior, mortality, weight gain, hematology and clinical hematological parameters (Thanabhorn et al., 2005). Mansoor et al. used a rapid and low-cost toxicity test (bioassay method for brine shrimp) to determine the toxicity of drugs compared with etoposide standard drug (LD50 = 7.4625). No obvious toxic effect was observed at concentrations of 10, 100 and 1,000 g/ml. The extracts from the aerial parts of different CR showed no acute toxicity at the dose of 1 g/kg b. w, and the body weight of mice was normal after 7 days of observation. Common side effects such as mild diarrhea, weight loss and depression were not recorded (Ahmad et al., 2012). In fact, mice showed significant anti-inflammatory and analgesic activities after oral administration of extracts from the aerial parts of CR up to 300 mg/kg b.w. (Soumaya et al., 2013). Singh et al. (2015) used acute oral administration of EECR in mice for 14 days, and the results showed no mortality at a dose of 2,000 mg/kg body weight. These results showed that the rhizome and tuber of CR and the aerial part of CR were safe and nontoxic. However, toxicological studies on other parts of CR, such as seeds, leaves, stems and bark, are necessary to determine the reliability and safety of different plant extracts, so further studies are needed.

## 7 Processing

Traditional Chinese medicine by processing can make the drug more pure, can remove the drug ingredients or impurities. Since Chinese medicinal materials are all the plants, animals or minerals that are collected from wild or cultivated and reared, or part of the natural state of dry products, often with other impurities, they must be separated and washed before processing, so as to achieve a certain purity and ensure the accuracy of clinical dosage. In addition, the properties of drugs can be changed or the curative effect of drugs can be enhanced by processing, and the toxicity of drugs can be weakened or eliminated by processing (Liu et al., 2022).

### 7.1 Processing method

In the Tang Dynasty, there was a method of stir-fry slightly (Lishang). In the Song Dynasty, steaming (Hongshi), boiling (Chuanxin), carbonizing (Jisheng fang). Fried with wine, roasted with licorice after soaking ginger juice, cooked with garlic after soaking rice swill, fried with lime, fried with vinegar after soaking children’s urine (Zhushi), etc. In the Yuan Dynasty, there were vinegar boiling (Huoyou), bran frying (Ruizhu) and other methods. In the Qing Dynasty, honey water frying and vinegar washing were added (Bencaoshu), human milk mix (Yaozhi)and other processing methods, more prominent is that the Ming and Qing Dynasties in the auxiliary materials system increased more. For example, Sizhi Xiangfu with wine, rice swill, children’s urine, salt water and wine, vinegar, children’s urine, salt after each soaking baking (Wanshi). Soaked with wine, vinegar, children’s urine, gardenia and stir fry (Zhunsheng), etc. The auxiliary material of Wuzhi Xiangfu are wine, vinegar, crisp, salt water and ginger juice (Dafa). Made of wine, vinegar, children’s urine, salt and ginger (Zhicai), etc. The auxiliary material of Liuzhi Xiangfu are rtemisia argyi, vinegar, salt, crisp, children’s urine, milk (Xingzhai), etc. The auxiliary material of Qizhi Xiangfu are children’s urine, yellow rice wine, vinegar, salt water, fennel soup, Yizhiren soup, radish soup, making finished baking (Diannan). One processing is for rice boiling bubbles, two processing is for aged wine bubbles, three processing is for children’s urine, four processing is for salt bubbles, five processing is for milk bubbles, six processing is for small flat black bean boiling, Qizhi Xiangfu is that Zhenfushen is ground into powder, making bolus with honey (Zengguang), etc. Bazhi Xianfu with wine, ginger, soil, vinegar, salt, children’s urine, milk successively processing (Shiyi), and other auxiliary materials, a total of nearly 50 kinds.Now the main processing methods are vinegar stir-fry, wine stir-fry, fried charcoal, etc (Table 4).

**TABLE 4:** The modern processing method of CR.

Processing method	Processing steps	Ref
Vinegar stir-fry	(1) Take the CR, add rice vinegar and mix them thoroughly, put them in a frying container, fry them to the prescribed degree, take them out and cool them. 20 kg rice vinegar for each 100 kg of CR.	Chinese Pharmacopoeia (2020 edition) Beijing
	(2) Take the original medicinal materials, remove hair and impurities, broken into pieces, add rice vinegar and mix well, moist for 1–2 h, until the rice vinegar is absorbed, put in a hot pot, stir-fry with gentle fire until the surface brown, take out, cool. 20 kg rice vinegar for each 100 kg of CR.	
Wine stir-fry	Mix the pieces or grains with rice wine until the rice wine is absorbed. Put them in a pot and fry them over a gentle heat of 110–120°C until they are scorched. Remove and cool. Each 100 kg of CR with grains or tablets, with 20 kg of rice wine.	Shandong
Fry charcoal	The pure CR with grain or pieces in a hot pot, big fire 180–220°C fry until the surface of the coke black, internal coke brown, spray a little water, put out the spark, take out, in time to air, cool thoroughly.	Shandong
Wine vinegar Stir-fry	Put the clean CR in the pot, add liquor and vinegar mixed solution, mix well, absorb. Stir-fry gently until the sections are brown to reddish brown, not scorched. Remove, let cool, sieve to remove crumbs, ready.	Yunnan
Wine vinegar salt honey Stir-fry	(1) Put the pure CR in a pot, add the liquor, vinegar, salt and the mixed water solution of refining honey, mix well, absorb, fry with gentle heat until the section is brown and not burnt. Remove, let cool, sieve to remove crumbs, ready. 40 g white wine, 100 g vinegar, 10 g salt and 20 g refined honey were used for every 1,000 g CR	Yunnan
	(2)Take clean CR, add rice vinegar, brown sugar, yellow rice wine, salt (brown sugar, salt dissolved in vinegar and yellow rice wine), mix well, absorb. Cook gently until brown. For every 100 kg of CR, use 100 kg of rice vinegar, 3 kg of brown sugar, 5 kg of yellow rice wine and 10 kg of salt.	Ningxia
	(3) Add vinegar, wine, salt water, brown sugar water mixture and appropriate amount of water to 2–3 cm of the medicine surface, cook for 8–10 h, braise overnight, cook again the next day until the medicine juice is absorbed, take out and dry. For each 100 kg piece of CR, use 20 kg vinegar, 2.5 kg salt, 5 kg brown sugar and 10 kg wine.	Hubei
Wine salt Stir-fry	Put the incense in a pot, add the water solution of salt and vinegar, mix well, and absorb. Stir fry gently until the sections are brown and not scorched. Remove, let cool, sieve to remove crumbs, ready. Use 100 g vinegar and 20 g salt for every 1,000 g CR.	Yunnan

### 7.2 Processing effect

CR as a traditional Chinese medicine, both can be used and processed. Processing can change the efficacy of CR, such as the raw CR can relieve exterior syndrome, and vinegar CR can dissipate pain, wine CR can dredge collaterals pain, CR after carbonization by stir-heating can stop bleeding. In clinical application, the raw CR upward chest septum, can reach limbs skin; cooked CR can enter the liver and kidney meridian for waist and foot; vinegar CR can enter the liver meridian, with blood stasis, convergence, detoxification, analgesia, dyspepsia; wine CR has the effect of meridians, can disperse stagnation, used to treat cold hernia abdominal pain; CR after carbonization by stir-heating taste bitter, warm, with hemostatic effect, more than treatment of female collapse; the SiZhiXiangFu processed by four kinds of excipients increased the effect of CR on qi stagnation and menstruation, which can be used to treat irregular menstruation, dysmenorrhea, rib swelling and pain.

Relieving dryness and increasing efficiency are generally considered as the purpose of CR processing. The ancients thought that wine, vinegar, rice swill and so on can slow down the dryness of raw CR. Modern studies mostly explain from the perspective of chemical composition. It is believed that the dryness of CR is due to its volatile oil composition. The volatile oil can be lost by heat volatilization during processing. The rice swill water is a starch suspension. The soaking and bleaching of CR can absorb some of its volatile oil, so that its content decreases and the dryness is alleviated (Jia, 1994). To weaken the stimulation of the gastrointestinal tract; after soaking CR in children’s urine, vinegar and wine, some polar or nonpolar components and alkaloids can be combined to form derivatives with weak irritation (Wu et al., 1989). The increasing efficacy of CR is generally believed to be related to the increase of hemostasis, dyspepsia, regulating qi and relieving depression, spasmolysis and analgesia (Yang 1991; Sun et al., 2007; Li et al., 2010; Liu et al., 2020). Many kinds of traditional Chinese medicine after charring, the effect of hemostatic and coagulation produced or enhanced, such as elm charcoal, sophora charcoal. CR after charring, also has coagulation and hemostatic effect. It is believed that calcium oxalate, the metabolic product originally existing in plants after carbonization of Chinese medicinal materials, is decomposed into calcium carbonate or calcium oxide after high-temperature calcination. After oral administration, soluble calcium salts that are easy to be absorbed are formed by gastric acid, which promotes the absorption of calcium and enhances the coagulation effect. At the same time, the adsorption of activated carbon can effectively stop bleeding after external application to the bleeding site (Yang 1979). CR after charring, can stop bleeding, commonly used in women more than collapse. A variety of elements were detected before and after carbonizing by stir-frying of CR. The results showed that the contents of Fe, Cr, Zn, Mg, Ca, Sr, Ba and P were the highest after carbonizing by stir-frying of CR, and the enhancement of hemostasis after carbonizing by stir-frying of CR might be related to the increase of Ca content (Jin et al., 1998).

Processing with vinegar is the main processing method of CR, which is divided into vinegar cooking, vinegar steaming, streaming with vinegar and vinegar stir-fry. The method of vinegar stir-frying CR is: take the CR granules or tablets, add quantitative vinegar to mix, slightly moist, after vinegar is absorbed, stir-frying container, heating with fire, stir-drying, take out, cool, sieve debris. The method of cooking CR with vinegar is: take the CR granules or slices, add quantitative rice vinegar to dry, slice, low temperature drying and screening. The method of vinegar steaming CR is: take pure CR, add quantitative rice vinegar and the same amount of water with rice vinegar, co-cook until vinegar is basically exhausted, and then steam for a moment, take out the cool, slice, dry, sieve debris. After vinegar stir-fry, it can enhance the effect of CR on soothing liver and relieving pain, and can dissipate stagnation. It is often used for abdominal pain, blood stagnation, cold coagulation, epigastric pain, etc. Vinegar stir-fry can increase the dissolution of total flavonoids from effective parts of CR (Li et al., 2011a). At present, some studies optimize the processing technology of processing with vinegar of CR by controlling various parameters, in order to obtain more reasonable processing technology. Because CR mainly contains volatile oil components, the changes of volatile oil caused by processing were studied in the literature. The results showed that the content of volatile oil decreased after processing, but the volatile oil was preserved more completely by vinegar stir-fry, so vinegar stir-fry was often used in clinical application (Sun et al., 2003; Li et al., 2011b).The yield of volatile oil from vinegar stir-fry CR was lower than that from raw CR, and the contents of various alkenes and ketones in the main components of volatile oil from vinegar stir-fry CR were higher than those in raw products. α-Cyperone can effectively inhibit the contraction of isolated uterine muscle and relieve fever and analgesia. After vinegar stir-fry, the content of α-Cyperone increased, which may be one of the material bases of increasing the effect of vinegar stir-fry (Sheng et al., 2013).

Modern pharmacological studies have shown that CR has analgesic antipyretic, antibacterial and anti-inflammatory effects, and its effective component is total saponins. The contents of total saponins and r-cyperone in CR were significantly increased after vinegar stir-fry. The results showed that vinegar stir-fry CR was beneficial to the increase of effective components and dissolution, thus enhancing the curative effect (Liu, 2014).After wine stir-fry, CR can pass through the meridians, scattered stagnation, more used for hernia pain, small intestine gas, and scrofula flow injection mass syndrome. The enhancement of curative effect may be related to the increase of total saponins. Some studies have shown that the content of total saponins in CR after vinegar stir-fry and wine stir-fry significantly increased, and the content after vinegar stir-fry increased significantly (Li et al., 2010).

The research on the pharmacodynamic material basis of traditional Chinese medicine has always been the key and difficult point in the research of traditional Chinese medicine, and CR is generally used after processing in clinic. The changes in drug properties after processing are bound to be accompanied by changes in chemical composition. CR mainly contains volatile oil, terpenes, flavonoids and other components. The process of processing can not only transform the components, but also change the proportion of each component. The change of medicinal properties is not necessarily the emergence of new components, but also the change of the proportion of each active component. Volatile oil is considered to be the most important pharmacodynamic material basis in CR, and α-Cyperone is generally considered to be an important effective component. In recent years, the pharmacological activities of other components have been gradually reported. The reported effective components areα-cyperone, cyperenone, sugeonol, Isolongifolene-5-one, caryophyllene oxide, ledene oxide- (II), spathulenol, stigmasterol, daucosterol, physcion, palmitic acid and so on. Most of them are terpenes (Li and Xie 2018; Chen et al., 2019; Yuan 2019; Wang et al., 2020; Yang 2021). In the process of processing, the composition transformation and content ratio change are very complex. The combination of quantitative and qualitative methods can be applied to the study of processing mechanism.

## 8 Pharmacokinetics

Guo et al. used HPLC method to study the pharmacokinetics of α-Cyperone in CR in rats. The results showed that T1/2 (h) and AUC_0−t_ (ng/ml h) were 10.70 ± 3.35, 2.13 ± 0.96 and 1,226.84 ± 346.82, 2,634.99 ± 571.25, respectively, after intragastric(ig) and intravenous injection administration of CR oil. The plasma concentration-time data of α-Cyperone in rats were fitted by 3P87, and the kinetic behaviors were in line with the two-compartment model. At the same time, the drug-time curve of ig administration measured in this study had three absorption peaks, and the peak time was 0.25, 2 and 12 h, respectively. The reasons for the multi-peaks may be as follows: α-Cyperone has a specific antagonistic effect on nifedipine-like calcium ions in voltage sensitive calcium channel. It is speculated that due to the decrease of peristalsis rate in rats, the ig administration time is prolonged and the absorption is irregular. The other reason is that CR has obvious choleretic effect on normal rats, which could promote bile secretion, increase bile flow, and increase the concentration of solids in bile. It is speculated that increased bile may promote the reabsorption of α-Cyperone in the gastrointestinal tract. (Guo and Meng 2009). However, traditional Chinese medicine extracts are often used in clinical treatment of related diseases. Traditional Chinese medicine believes that there are many active ingredients in Chinese medicine, which interact with each other and act on multiple targets. However, this study only studied monomer compounds, lacking scientificity and reliability.

At present, there are few studies on pharmacokinetics of CR, which may be due to the variety and complexity of chemical constituents. In addition, patients often give different doses of CR according to age, gender, weight, physiological differences and other factors. At present, there is no pharmacokinetic study of different doses of CR *in vivo*. In addition, processed products of CR are often provided to patients in clinical practice. In future research, we should pay attention to the comparison of original and processed products of CR.

## 9 Analytical methods and quality control

At present, Chinese Pharmacopoeia (2020 edition) uses α-Cyperone as the quality control index of CR, and uses thin layer chromatography (TLC) for qualitative identification and HPLC for quantitative identification. The content of volatile oil in this product should not be less than 1.0%. But this method takes a long time to analyze. The solvent consumption is high, and the perfume contains many kinds of components, different components work together, only one or several effective components as indicators to evaluate the quality of perfume is not scientific and objective.

In addition, there are many analytical methods for chemical constituents of CR. For example, Wu et al. used NaNO_2_-Al(NO_3_)_3_-NaOH colorimetric method to determine the total flavonoids content in CR, which provided a reference for the establishment of the determination method of total flavonoids content in CR (Yun et al., 2014). Cao et al. established the UV method of total flavonoids in CR, and investigated the content of total flavonoids in CR from different habitats, to provide the basis for the quality control of CR. This method is simple, accurate, and can be used for the quality control of CR. This method can be used for the analysis of compounds in CR with good separation effect and good stability (Mei and Lu 2015). However, it is not suitable for compounds with no ultraviolet absorption or only terminal absorption. Liang et al. used HPLC to determine the content of α-Cyperone in the oil of CR extracted with supercritical CO_2_ (Liang et al., 2004). The method is rapid and accurate, making it a good choice for quality control in CR. Deng et al. established the UHPLC method for the determination of mesocyperusphenol A, scirpusins A and cyperusphenol A in CR, which provided the basis for the reasonable quality control of CR (Deng et al., 2015a; Deng et al., 2015b). Compared with the HPLC detection technology, the UHPLC greatly improves the number of column trays due to the use of smaller particle size (sub 2 μm) chromatographic packing, thereby enhancing the chromatographic separation ability and completing the analysis of samples in a shorter time. At the same time, it has a series of advantages such as less sample consumption, solvent saving and high sensitivity. The volatile oil compounds of CR were detected by GC-MS. Eman Ahmed et al. analyzed 44 kinds of volatile oil components by GC-MS (El-Wakil et al., 2019; Lu et al., 2021). Lin et al. analyzed 45 volatile oil components in CR by GC-MS (Xiao-shan et al., 2006). Some scholars also used Head Space/Solid Phase Micro-Extraction coupled with Gas Chromatography/Mass Spectrometer (HS-SPME-GC/MS) and Liquid Chromatography coupled to quadrupole time of flight-mass spectrometry (LC-QTOF-MS) to analyze the chemical constituents of CR. The method has high sensitivity and strong anti-interference ability, but the equipment is expensive (He et al., 2018; Zhou et al., 2019; Qiu et al., 2021).In addition, some scholars analyzed TOF of CR by LC-ESI-MS/MS method (Kandikattu et al., 2015).

Most of these methods have the advantages of fast, precision, high accuracy, high sensitivity, short analysis time, strong separation ability, good selectivity, simple operation, low detection limit and wide application range. However, most methods have shortcomings such as complex sample pretreatment, high-end equipment, high price, toxic organic solvents, environmental soil pollution, and insufficient green environmental protection. But we believe that with the development of science and technology, they will have more and more low-cost, efficient instruments and environmentally friendly green reagents.

At present, Chinese Pharmacopoeia (2020 edition) uses α-Cyperone as the quality control index of CR, and uses TLC for qualitative identification and HPLC for quantitative identification. The content of volatile oil in this product should not be less than 1.0%. But this method analysis time is long, the solvent consumption is big, and the perfume contains many components, different components work together, only one or several effective components as indicators to evaluate the quality of perfume will exist some problems cannot be ignored. Traditional Chinese medicine believes that there are many active ingredients in traditional Chinese medicine. They interact and act on multiple targets. The chemical composition of CR is complex. It is not enough to evaluate the quality of CR only by the content of several compounds. Therefore, in order to evaluate the quality of CR, we can refer to the above methods. Future researchers should establish a spectral-effect relationship method to screen active compounds, or establish a network pharmacology method to predict compounds related to diseases or disease targets, and then verify them through *in vitro* and *in vivo* studies. We strongly suggest that future experiments should be clinically verified to confirm the clinical safety and effectiveness of this plant, so as to develop new therapeutic strategies for the prevention and treatment of disease, and finally develop new drugs suitable for clinical application.

## 10 Conclusion and prospect

In this paper, the research progress of CR in botany, traditional application, phytochemistry, pharmacology and so on are reviewed. At the same time, the analysis method, quality control, processing method and processing are summarized and analyzed. It will be helpful for future scholars to further study the CR. In addition, this paper also has an in-depth discussion on the shortcomings of the current researches in some aspects of CR, and put forward our own views and solutions:

First, at present, the researches on the volatile oil components are mainly focused on, with more in-depth studies and great research results achieved. However, there are few studies on other chemical constituents and their pharmacological effects. In the future scholars can conduct more and more in-depth researches on other components to provide theoretical support for the further development and utilization of modern CR and improve the medicinal value of CR. At the same time, the studies of effective substance of CR are not in-depth, and a large number of studies remain in the extract stage, and the mechanism, target and pathway of action of the active ingredients on the disease have not been scientifically clarified. Scholars can consider using network pharmacology, virtual screening and other technologies to predict the chemical constituents and pharmacological activities related to targets, receptors and pathways, and verify them through pharmacological experiments. In addition, new data mining methods can be proposed to screen herbs with similar effects, thus making more effective use of natural medicines and reducing the cost of new drug development.

Second, CR of vinegar stir-frying are often used in clinical treatment, so the different extraction methods and different processing methods play an important role in the role of CR. This suggests, when it is applied in clinical treatment to the attached to its corresponding extraction method and processing technique should be paid attention in the further at the same time, we should also recognize that our researches on the chemical composition of CR and its clinical treatment related links and mechanisms of action is not enough, and its pharmacological effects in the scope of still need to be further explored. In the future, researchers can further explore the effective chemical components of CR and continue to study its potential medicinal value, so that it can play a greater role in clinical, and constantly promote the development of Traditional Chinese Medicine.

Third, to ensure the safety and efficacy of drugs, toxicity assessment can provide guidance for clinical efficacy and patient safety. At the same time, toxicity evaluation is also a prerequisite for new drug development. However, there are few reports of CR on toxicity evaluation and pharmacokinetics of main active components *in vivo*. In addition, scholars should study the toxicity and pharmacokinetic characteristics of the compounds in CR, so as to provide experimental basis for the new drug development of CR.

Fourth, from ancient times, there are many processing methods of CR, such as Sizhi Xiangfu, wine CR, CR charcoal, etc. Different processing methods have different performance and usage. But now the processing method recorded in the pharmacopoeia is only vinegar stir-frying, so it is necessary to further study the clinical efficacy of different processed products of CR and further clarify the relevant processing mechanism.

This review makes up for the deficiencies of previous reviews of CR in the aspects of analytical methods, quality control, processing methods, processing functions and pharmacokinetics, and discusses the limitations of current studies. In order to provide a valuable reference for the future scholars to develop and utilize the CR.

## Glossary

ACA: attar extracted from CR
BSA: bovine serum albumin
CAT: catalase
CR: Cyperi Rhizoma
DPPH: 1,1-diphenyl-2-picrylhydrazine
ECR: effects of rhizome extract
EECR: ethanol extract of CR
FRAP: Ferric ion reducing antioxidant power
FST: forced swimming test
GC-MS: gas chromatography-mass spectrometry
GO: gene ontology
GSH-PX: glutathione peroxidase
HDL-C: high density lipoprotein cholesterol
HO-1: heme oxygenase-1
HPLC: high-performance liquid chromatography
iNOS: inducible nitric oxide synthase
KEGG: Kyoto gene and genome encyclopedia
LDL-C: low density lipoprotein cholesterol
LPS: lipopolysaccharide
MAO: Monoamine oxidize A
MDD: Ver extract isolated from CR
MRCr: methanol extract of CR
NO: nitric oxide
OFT: open field test
PGE2: prostaglandin E2
PID: pelvic inflammatory disease
ROS: reactive oxygen species
SOD: superoxide dismutase
STZ: streptozotocin
TC: total cholesterol
TG: triglyceride
TLC: thin layer chromatography
TOF: total oligoflavones
TST: tail suspension test
VEGF: vascular endothelial growth factor
XFE: the 95% ethanol extract isolated from CR
XFW: the 95% water extract isolated from CR
