# Supplementary material for: Systematic review of ethnomedicine, phytochemistry, and pharmacology of Cyperi Rhizoma
Source: Front Pharmacol. 2022 Oct 7;13:965902. doi: 10.3389/fphar.2022.965902 (PMC9585201; doi:10.3389/fphar.2022.965902)
Supplement: Supplementary file 1 [file Table1.DOCX]

**Table A1** Identification of genuine and counterfeit characters of CR.

| **Name** | **Source** | **Characters** | **Four qi and five flavors** |
| --- | --- | --- | --- |
| Cyperi Rhizoma (quality goods) | Dry rhizomes of Cyperaceae plants | It is spindle-shaped, some slightly curved, 2 ~ 3.5 cm in length and 0.5 ~ 1 cm in diameter. The surface was brown or dark brown, with longitudinal wrinkles and 6 ~ 10 slightly uplifted links. There were unextinct brown hairy whiskers and broken roots on the joints. The hair removal was smooth and the link was not obvious. Hard, boiled section yellow brown or red brown, cuticle ; the cross-section of sunburners was white and powdery, the inner skin was ring-shaped, the middle column color was dark, and the dotted vascular bundles were scattered. | Fragrance, bitter taste. |
| Cyperus stoloniferus(ersatz goods) | Coarse rhizomes of Cyperaceae plants.  Dry rhizomes of sedge. | Length 2-5 cm, diameter 0.5-1.5 cm. Surface brown or dark brown. The links are obvious, and the nodes are dense, especially at both ends. There are many links, more than 6-12, and a few can reach 35. Light and hard texture, section light brown or red brown. | Fragrance, bitter and hard. |
| rhizoma anemones raddeanae | Dry rhizomes of Anemone raddeana Regel | Long spindle-shaped, slightly curved, generally slender, some with short branches, links are not obvious, hairless fibers. Smooth surface, brown to dark brown. | Faint breath, bitter taste. |
