# Supplementary material for: Systematic review of ethnomedicine, phytochemistry, and pharmacology of Cyperi Rhizoma
Source: Front Pharmacol. 2022 Oct 7;13:965902. doi: 10.3389/fphar.2022.965902 (PMC9585201; doi:10.3389/fphar.2022.965902)
Supplement: Supplementary file 2 [file Table2.docx]

**Table A2** The others clinical prescriptions of CR

| Prescription name | Main components | Formulation | Traditional and clinical uses | Reference |
| --- | --- | --- | --- | --- |
| Buxue Dingtong Tang | Angelica sinensis  Radix rehmanniae praeparata  Rhizoma corydalis  Safflower carthamus  CR | Decoction | Promote blood circulation, relieve pain, remove blood stasis, postpartum stasis and abdominal pain. | Wan Bing Hui Chun  (万病回春) |
| Gujing Tang | Cortex phellodendri  Radix paeoniae alba  Scutellaria baicalensis  CR  Sanguisorba officinalis  Astragalus membranaceus | Decoction | Clear heat and cool blood, promote blood circulation and remove stasis, invigorate qi and solidify the foundation, nourish blood and stop bleeding. | Song Ya Zun Sheng(嵩崖尊生) |
| Shentong Zhuyu Tang | Radix gentianae macrophyllae  Ligusticum wallichii  Trogopterus dung  CR | Decoction | Circulation of blood qi, dispel wind dehumidification, bi pain relief. | Yi Lin Gai Cuo (医林改错) |
| Aifu Nangong Wan | Folium artemisiae argyi  CR  Cassia twig  Angelica sinensis | Pill | Warm meridian nourish blood warm uterus. | Shen Shi Zun Sheng Shu  (沈氏尊生书) |
| Qingru Wan | CR  Malt  Pericarpium citri reticulatae  Fructus amomi | Pill | Warm and fast diaphragm, stop vomiting, eliminate breast food. | Ying Tong Bai Wen(婴童百问) |
| Xuanyu Tongjing Tang | Radix paeoniae alba  Angelica sinensis  Cortex moutan  Radix bupleuri  CR | Decoction | Women with irregular menstruation, chronic pelvic inflammatory disease, endometritis, functional uterine bleeding. | Fu Qing Zhu Nv Ke(傅青主女科) |
| Anlao Tang | Ginseng  Rhizoma atractylodis macrocephalae  Angelica sinensis  CR | Decoction | Nourish spleen and liver, nourish Yin and stop leakage. | Fu Qing Zhu Nv Ke(傅青主女科) |
| Antai Fuyuan Yin | Fructus aurantii  CR  Rhizoma atractylodis macrocephalae  Salvia miltiorrhiza | Decoction | Miscarriage prevention | Zheng Shi Jia Chuan Nv Ke Wan Jin Fang(郑氏家传女科万金方) |
| Antai Gao | Rhizome of rehmannia  Ligusticum wallichii  Eucommia ulmoides  Scutellaria baicalensis  CR | Ointment | Prevent miscarriages | Li Yue（理瀹） |
| Xiangfu Wan | CR  Angelica sinensis  Ligusticum wallichii  Radix paeoniae alba  Radix rehmanniae praeparata  Rhizoma atractylodis macrocephalae  Fructus amomi  Pericarpium citri reticulatae  Scutellaria baicalensis | Pill | Soothe liver and spleen, nourish blood and regulate menstruation. | Chinese Pharmacopoeia |
| Anxiang Tang | CR  Fructus amomi  Radix aucklandiae  Santalum album  Licorice | Decoction | Refreshing | Shi Zhai Bai Yi Xuan Fang([是斋百一选方](http://www.zysj.com.cn/lilunshuji/shizhaibaiyixuanfang/)) |
| Babao Ruisheng Dan | Angelica sinensis  Poria cocos  Rhizoma zingiberis  Katsumadai seed  Radix curcumae  CR | Pill | Open the stomach. Heart and stomach pain, chest tightness and abdominal distension, acid swallowing and vomiting, dyspepsia, qi discomfort, chest and flank pain. | Quan Guo Zhong Yao Cheng Yao Chu Fang Ji(全国中药成药处方集) |
| Baibai Wan | Rhizoma atractylodis macrocephalae  Cortex phellodendri  Rhizome of rehmannia  Radix paeoniae alba  CR | Pill | Blood under hot and humid. | Yi Xue Ru Men(医学入门) |
| Baizi Fugui Wan | Folium artemisiae argyi  Angelica sinensis  Ligusticum wallichii  Radix rehmanniae praeparata  CR | Pill | Regulating menstruation and nourishing blood, placenta and qi. | She Shneg Zhong Miao Fang(摄生众妙方) |
| Banxia Houpu Tang | Pinellia ternata  Mangnolia officinalis  Cape jasmine  Coptis chinensis  Atractylodes lancea  CR | Decoction | Stomach-churning, spitting, chest pain. | Zhi Zhi Fu Yi(直指附遗) |
| Baochan Siwu Tang | Angelica sinensis  Radix paeoniae alba  Herba leonuri  CR | Decoction | Postpartum is deficient. | Lu Fu Jin Fang(鲁府禁方) |
| Binglang Sixiao Wan | Areca catechu  Fructus aurantii immaturus  Hawthorn  Radix aucklandiae  Mangnolia officinalis  CR | Pill | Digesting water, chest and abdomen distension. | Bei Jing Shi Zhong Cheng Yao Fang Xuan Ji(北京市中药成方选集) |
| Bizheng Tang | CR  Angelica sinensis  Ligusticum wallichii  Aconite | Decoction | Arthralgia | Mai Zheng Zheng Zong(脉症正宗) |
| Baisheng Dafuoshou Tang | Angelica sinensis  Ligusticum wallichii  Eucommia ulmoides  Licorice  CR | Decoction | Big tonic qi and blood, qi and pain relief. | Chen Su An gynecology tonic solution（陈素庵妇科补解） |
| Yuxuebi Keli | Olibanum  Myrrh  Safflower carthamus  Radix clematidis  Radix achyranthis bidentatae  CR | Granule | Promoting blood circulation and removing blood stasis, removing collateral and relieving pain are used for bi disease caused by blood stasis. | Chinese Pharmacopoeia |
| Shuerjing Keli | Angelica sinensis  Radix paeoniae alba  Radix paeoniae rubra  CR  Rhizoma corydalis | Granule | Promote blood circulation, relieve pain and regulate menstruation. Used for dysmenorrhea. | Chinese Pharmacopoeia |
| Jintongxiao Ding | Olibanum  Kaempferia rotunda  Ligusticum wallichii  Kadsura root-bark  Angelica dahurica  CR | Tincture | Promote blood circulation, remove blood stasis, reduce swelling and relieve pain. For acute closed soft tissue injury. | Chinese Pharmacopoeia |
| Qinghou Liyan Keli | Scutellaria baicalensis  Platycodon grandiflorum  Sterculia lychnophora  Fructus aurantii  CR  Menthol | Granule | Clear heat benefit pharynx, wide chest moisten throat. Used for dry throat and hoarseness caused by external wind heat; Acute and chronic pharyngitis, tonsillitis. | Chinese Pharmacopoeia |
| Dusheng Huoxue Pian | Kaempferia rotunda  CR  Angelica sinensis  Rhizoma corydalis  Caulis spatholobi | Tablet | Promote blood circulation, reduce swelling, regulate qi and relieve pain. Used for dyspenorrhea caused by bruises, blood stasis and swelling and qi stagnation and blood stasis. | Chinese Pharmacopoeia |
| Xiaozheng Wan | Radix bupleuri  CR  Rheum officinale  Pericarpium citri reticulatae viride  Curcuma zedoary | Pill | It is used for the mammary gland enlargement caused by qi stagnation, blood stasis and spittoon. | Chinese Pharmacopoeia |
| Tongbi Pian | Strychnos nux-vomica  Centipede  Buthus martensi karsch  Lumbricus  Gastrodia elata  Ginseng  Peach kernel  The seed of cowherb  Caulis spatholobi  CR | Tablet | Dispel wind and win dampness, promote blood circulation and dredge collaterals, disperse cold and relieve pain, regulate and tonic qi and blood for bi disease caused by cold and dampness blocking blood stasis blocking collaterals qi and blood deficiency. | Chinese Pharmacopoeia |
| Nvjin Jiaonang | Angelica sinensis  Ligusticum wallichii  Codonopsis pilosula  Poria cocos  Herba leonuri  Angelica dahurica  CR | Capsule | To invigorate qi and nourish blood, regulate qi and invigorate blood, and relieve pain for irregular menstruation caused by qi and blood deficiency. | Chinese Pharmacopoeia |
| Lemai Jiaonang | Salvia miltiorrhiza  Ligusticum wallichii  Radix paeoniae rubra  Safflower carthamus  CR  Radix aucklandiae | Capsule | Promote qi and blood circulation, remove blood stasis and pass the pulse. For headache, vertigo, chest pain and palpitations caused by qi stagnation and blood stasis; Coronary heart disease, angina pectoris, multiple cerebral infarction. | Chinese Pharmacopoeia |
| Houtou Jianweiling Pian | Hericium mycelium  Cuttlebone  Rhizoma corydalis  Radix paeoniae alba  CR  Licorice | Tablet | Soothe liver and stomach, regulate qi and relieve pain. Used for liver and stomach disharmony, stomachache, vomiting and acid swallowing; Chronic gastritis, gastric and duodenal ulcer. | Chinese Pharmacopoeia |
| Tiaojing Huoxue Jiaonang | Radix aucklandiae  Ligusticum wallichii  Rhizoma corydalis  Angelica sinensis  Radix paeoniae rubra  Safflower carthamus  CR | Capsule | Nourish blood and promote blood circulation, perform qi and relieve pain for irregular menstrual dyspenorrhea caused by qi stagnation and blood stasis and blood deficiency. | Chinese Pharmacopoeia |
| Xiaoyukang Pian | Angelica sinensis  Ligusticum wallichii  Radix achyranthis bidentatae  Safflower carthamus  CR | Tablet | Promote blood circulation, remove blood stasis, reduce swelling and relieve pain. For the treatment of intracranial hematoma absorption period. | Chinese Pharmacopoeia |
| Ershiqiwei Dingxiao Wan | American ginseng  Poria cocos  Astragalus membranaceus  Schisandra chinensis  Cornua cervi pantotrichum  Folium artemisiae argyi  Mangnolia officinalis  CR | Pill | Tonifying qi and nourishing blood, relieving depression and regulating menstruation, used to flush the deficiency of ren, deficiency of both Qi and blood, emassive body, irregular menstruation, menstrual disorder, abdominal pain, continuous leakage, sour back and weak legs. | Chinese Pharmacopoeia |
| Qizhi Weitong Keli | Radix bupleuri  Rhizoma corydalis  Fructus aurantii  CR  Licorice | Granule | Soothe liver and qi, and relieve stomach pain for liver qi stagnation, chest bulges and fullness, epigastric pain. | Chinese Pharmacopoeia |
| Shenwu JiannaoJiaonang | Ginseng  Polygonum multiflorum  Polygala tenuifolia  Scutellaria baicalensis  Spina date seed  CR | Capsule | Tonifying kidney and filling essence, supplementing qi and nourishing blood, strengthening the body and strengthening the brain are used for mental exhaustion caused by deficiency of kidney essence, deficiency of liver qi and blood, insomnia, dreaming, dizziness and dizziness, physical weakness and memory loss. | Chinese Pharmacopoeia |
| fufang Yigan Wan | Herba artemisiae Gentian The dandelion  Selfheal  CR | Pill | Clearing heat and relieving dampness, soothing liver and spleen, removing blood stasis and dispersing knot. It is used for swelling pain of flank, jaundice, dry mouth, bitter moss and yellow vein string caused by dampness and heat. | Chinese Pharmacopoeia |
| Weisu Keli | Perilla stem  CR  Pericarpium citri reticulatae  Fingered citron  Fructus aurantii | Granule | Qi regulating and eliminating distension, and stomach analgesia are mainly used to treat qi stagnation type epigastric pain. Symptoms include epigastric distension pain, channeling and two flanks, belching or qi relieving, emotional depression and anger aggravating, chest distress, less food, poor defecation, thin white tongue coating, and string pulse.Chronic gastritis and peptic ulcer. | Chinese Pharmacopoeia |
| Weiyangning Wan | Rhizoma atractylodis macrocephalae  Lindera aggregata  Yam Galangal  CR | Pill | 温中散寒，理气止痛，制酸止血。用于胃脘胀痛或刺痛，呕吐泛酸，胃及十二指肠溃疡 | Chinese Pharmacopoeia |
| Qiwei Putao San | White raisins  Safflower carthamus  CR  Pomegranate | Podwer | Warm and dissipate cold, regulate qi and relieve pain, make acid to stop bleeding for epigastric distension or tingling pain, vomiting pantothenic acid, gastric and duodenal ulcer. | Chinese Pharmacopoeia |
| Wuji Baifeng Wan | Silkie  Antler glue Turtle shell  Ginseng  Radix paeoniae alba  CR | Pill | Tonifying qi and nourishing blood, regulating menstruation and stopping band is used for deficiency of qi and blood, emasculation, waist and knees, irregular menstruation, and leakage band. | Chinese Pharmacopoeia |
| Fufang Niuhuang Qingwei Wan | Rheum officinale  Petunia Gardenia Glauber's salt  Coptis chinensis  Forsythia suspensa  Mangnolia officinalis  CR | Pill | Clearing heat and reducing fire, detoxifying and relieving constipation for sore of the tongue caused by gastrointestinal heat, gingival swelling, pain, pharyngeal diaphragm, constipation, short and red urine. | Chinese Pharmacopoeia |
| Zhonghua Dieda Wan | Cattle cane  Nostoc commune  Oxtail dish  Radix linderae  Bee sage  Caulis spatholobi  Butyl tomato root Radix angelicae pubescentis  Atractylodes lancea  Monkshood  CR | Pill | Relieve swelling and pain, relax tendons and activate collateral, stop bleeding and produce muscle, promote blood circulation and remove blood stasis for bruising muscles and bones, old and new blood stasis, wound bleeding, rheumatism and blood stasis. | Chinese Pharmacopoeia |
| Awei Huapi Gao | CR  Mangnolia officinalis  Trigone  Curcuma zedoary  Angelica sinensis  Garlic | Ointment | It can be used for qi stagnation and blood coagulation, lump syndrome, abdominal pain, chest enlargement. | Chinese Pharmacopoeia |
| Baicaoxiang Jieyu Anshen Jiaonang | Selfheal  Radix bupleuri  Caulis polygoni multiflori  Radix paeoniae alba  CR  Flos albiziae Fruit of Chinese magnoliavine | Capsule | Soothing the liver, relieve depression, calm the mind for insomnia is a liver qi stasis syndrome, insomnia symptoms do not relieve the chest flank distension or pain, bitter abdominal distension pulse string. | Chinese Pharmacopoeia |
