# Supplementary material for: Systematic review of ethnomedicine, phytochemistry, and pharmacology of Cyperi Rhizoma
Source: Front Pharmacol. 2022 Oct 7;13:965902. doi: 10.3389/fphar.2022.965902 (PMC9585201; doi:10.3389/fphar.2022.965902)
Supplement: Supplementary file 3 [file Table3.docx]

Table A3. The patent application status of CR.

| Proprietary name | Patent number |
| --- | --- |
| A plant additive for pig feed and its preparation and application | CN201711270235.2 |
| A Chinese herbal compound for reducing intramuscular fat in chicken and its preparation method | CN202010248271.4 |
| A formula of nourishing stomach and invigorating spleen five grain soybean juice | CN201910373270.X |
| A fermented flour food preservative, preparation method and application method | CN201711235200.5 |
| A health wine to prevent female sub-health | CN202011500068.8 |
| A clean cream and its preparation method | CN202010974552.8 |
| A thyme medicinal tea and its preparation method and application | CN202110406590.8 |
| A herbal koji and its preparation method | CN202110800811.X |
| A clean gingival care tooth powder and its preparation method | CN202110141429.2 |
| A mosquito repellent bale and its preparation method | CN202011279657.8 |
