# Supplementary material for: Systematic review of ethnomedicine, phytochemistry, and pharmacology of Cyperi Rhizoma
Source: Front Pharmacol. 2022 Oct 7;13:965902. doi: 10.3389/fphar.2022.965902 (PMC9585201; doi:10.3389/fphar.2022.965902)
Supplement: Supplementary file 4 [file Table4.docx]

Table A4. Chemical constituents of CR that have been isolated and identified.

| No. | Compounds | Reference |
| --- | --- | --- |
| volatile oil | | |
| 1 | camphene | [1] |
| 2 | β-pinene | [1] |
| 3 | limonene | [1] |
| 4 | 1,8-cineole | [1] |
| 5 | γ-cymene | [1] |
| 6 | β-selinene | [2] |
| 7 | selinatriene | [2] |
| 8 | cyperol | [2] |
| 9 | isocyperol | [2] |
| 10 | α-cyperone | [1] |
| 11 | β-cyperone | [1] |
| 12 | α-rotunol | [2] |
| 13 | β-rotunol | [2] |
| 14 | 4α,5α-oxidoeudesm-11-en-3α-ol | [3] |
| 15 | 11-eremo-philadien-2-one( nootkatone) | [4] |
| 16 | eudesma-4 | [5] |
| 17 | 11-dien-3β-o1 | [5] |
| 18 | nootkatene | [6] |
| 19 | α-selinene | [6] |
| 20 | epi-α- selinene | [6] |
| 21 | valencene | [6] |
| 22 | 4,7-dimethyl-1-tetralone, | [7] |
| 23 | 10,12-peroxycalamenene | [8] |
| 24 | calamenene | [8] |
| 25 | trans-calamenene | [8] |
| 26 | cadalene | [8] |
| 27 | δ-cadinene | [8] |
| 28 | α-muurolene | [6] |
| 29 | γ- muurolene | [6] |
| 30 | γ-calacorene | [6] |
| 31 | cyperotundone | [9] |
| 32 | cyperene | [1] |
| 33 | sugetriol | [10] |
| 34 | patchoulenone | [1] |
| 35 | isopatchoula-3,5-diene | [11] |
| 36 | sugeonol | [12] |
| 37 | sugetriol triacetate | [5] |
| 38 | sugeonyl acetate | [8] |
| 39 | patchoulenyl acetate | [8] |
| 40 | cypera-2,4-diene | [6] |
| 41 | (-)-cypera-2,4(15)-diene | [6] |
| 42 | sugebio1 | [12] |
| 43 | cyperenoic acid | [13] |
| 44 | rotundone | [14] |
| 45 | epoxy-quaine | [14] |
| 46 | isocurcumenol | [15] |
| 47 | guaidiol | [13] |
| 48 | epi-guaiol | [13] |
| 49 | caryophyllene | [16] |
| 50 | isokobusone | [17] |
| 51 | kobusone | [17] |
| 52 | caryophyllene-6,7-oxide(β-caryophyllene 6,7-oxide) | [18] |
| 53 | caryophyllene-α-oxide | [18] |
| 54 | carophy1la-6-one | [7] |
| 55 | copadiene | [14] |
| 56 | copaene | [19] |
| 57 | mustakone | [19] |
| 58 | rotundene | [20] |
| 59 | rotundenol | [20] |
| 60 | (-)-norrotundene | [6] |
| 61 | (-)-isorotundene | [6] |
| 62 | α-humulene | [21] |
| 63 | β-elemene | [21] |
| 64 | (+)-cyperadione | [6] |
| 65 | 5-secoeudesmane | [4] |
| 66 | aristolone | [15] |
| 67 | cyclic acetal | [4] |
| 68 | cyperolone | [22] |
| 69 | (-)-clovane-2,9-diol | [23] |
| 70 | norcyperone | [23] |
| 71 | 6-hydroxy-3,5,11-cinetriene-2-one | [24] |
| 72 | (10R)－13－noreudesma－4,6－dien－3,11－dione | [24] |
| 73 | (4S,5E,10R)-7-oxo-tri-noreudesm-5-en-4β-ol | [25] |
| 74 | 4-hydroxy-4,7-dimethyl-1-tetralinone | [25] |
| 75 | 6-methyl-2-isopropenyl-7,10-dioxo undecanoic acid | [25] |
| 76 | 4α,5α-Oxidoeudesm- 11-en-3-one | [26] |
| 77 | cyper-11-ene-3,4-dione | [26] |
| 78 | rotunduside | [27] |
| 79 | cyperalin A | [28] |
| 80 | sugetriol triacetate | [28] |
| 81 | cyperene-3,8-dione | [29] |
| 82 | 14-hydroxycypero-tundone | [29] |
| 83 | 14-acetoxycyperotundone | [29] |
| 84 | 3β-hydroxycypere-noicacid | [29] |
| 85 | sugetriol-3,9-diacetate | [29] |
| 86 | rotunduside G | [30] |
| 87 | rotunduside H | [30] |
| 88 | epi-guaidiol A | [13] |
| 89 | rotundusolide A | [31] |
| 90 | rotundusolide B | [31] |
| 91 | ishwarol B | [32] |
| 92 | chrysanthemumin C | [32] |
| flavonoids | | |
| 93 | rhamnetin3-O-rhamnosyl-(1→4)-rhamnopyranoside | [33] |
| 94 | leucocyanidin | [34] |
| 95 | quercetin | [35] |
| 96 | chrysoeriol | [35] |
| 97 | luteolin | [35] |
| 98 | kaempferol | [35] |
| 99 | quercetin-3- 0-β-D-rutinoside | [35] |
| 100 | pinoquercetin | [36] |
| 101 | amentoflavone | [36] |
| 102 | bibbetin | [36] |
| 103 | ginkgetin | [36] |
| 104 | isog inkgetin | [36] |
| 105 | sciadopitysin | [36] |
| 106 | α-cyperone | [37] |
| 107 | 6-O-p-hydroxybenzoyl-6-epi-aucubin | [37] |
| 108 | 6-O-p-hydroxy-benzoyl-6-epi-monomelittoside | [37] |
| 109 | syringopicroside B | [37] |
| 110 | syringopicroside C | [37] |
| 111 | oleuropeinic acid | [37] |
| 112 | oleuroside | [37] |
| 113 | 10-hydroxyoleuropein | [37] |
| 114 | senburiside I | [37] |
| 115 | 4'-methoxy-8-methoxy-7-γ | [38] |
| 116 | 7-methoxyisoflavone | [38] |
| 117 | 5-hydroxy-4’-methoxy-7-［（3-methyl-2-buthenyl）oxy］-isoflavone | [38] |
| Triterpenes and sterols | | |
| 118 | β-sitosterol | [39] |
| 119 | oleanolic acid | [40] |
| 120 | β-sitosterol glucoside | [41] |
| 121 | stigmasterol glucoside | [41] |
| 122 | stigmasterol | [41] |
| 123 | 5α,8α-epicondioxy-(20S,22E,24R)-ergosterol-6,22-diene-3β-ol | [23] |
| 124 | sitosteryl(6-hentriacontanoyl)-β-D-galactopyranoside | [42] |
| 125 | taraxerone | [25] |
| 126 | dammaradienyl acetate | [25] |
| 127 | zeorin | [25] |
| Alkaloid | | |
| 128 | sarmentine | [25] |
| 129 | guineensine | [25] |
| 130 | pellitorine | [25] |
| 131 | caprolactam | [25] |
| 132 | Rotundine A | [43] |
| 133 | Rotundine B | [43] |
| 134 | Rotundine C | [43] |
| Others | | |
| 135 | rosenonolactone | [23] |
| 136 | sucrose | [44] |
| 137 | D-fructose | [45] |
| 138 | D-glucose | [45] |
| 139 | oxyphyllenone C | [46] |
| 140 | Cyprotuoside C | [47] |
| 141 | Cyprotuoside D | [47] |
| 142 | 1α-methoxy-3β-hydroxy-4α-(3′,4′-dihydroxyphenyl)-1,2,3,4-tetrahydronaphthalin | [48] |
| 143 | 1α,3β-di-hydroxy-4α-(3′,4′-dihydroxyphenyl)-1,2,3,4-tetrahydronaphthalin | [48] |
| 144 | emodin methyl ether | [49] |
| 145 | Catenarin | [49] |
| 146 | liriodendrin | [25] |

1. Trivedi, B., et al., *On terpenes. CLXIV. Composition of the oil from Cyperus rotundus L. structure of patchoulenone.* 1964. **29**(7): p. 1675-1688.

2. Hikino, H., et al., *Structure and absolute configuration of cyperol and isocyperol.* 1967. **15**(12): p. 1969.

3. Hikino, H. and K. Aota, *4α,5α-Oxidoeudesm-11-en-3α-ol, sesquiterpenoid of Cyperus rotundus.* 1976. **15**(8): p. 1265-1266.

4. Ohira, S., et al., *Sesquiterpenoids from Cyperus rotundus.* 1998. **47**(8): p. 1577-1581.

5. Wen, D., R. Zhang, and S.J.B.D.X.X.B. Chen, *Separation of chemical components in Cyperus rotundus rhizomes and its effects on ovary muscular contraction of uncyophoric rats in vitro.* 2003. **35**: p. 110-111.

6. Sonwa, M.M. and W. Nig, *Chemical study of the essential oil of Cyperus rotundus.* 2001. **58**(5): p. 799-810.

7. Thebtaranonth, C., et al., *Antimalarial sesquiterpenes from tubers of Cyperus rotundus : structure of 10,12-Peroxycalamenene, a sesquiterpene endoperoxide.* 1995. **40**(1): p. 125-128.

8. Komai, K. and C.-S.J.P. Tang, *A chemotype of Cyperus rotundus in Hawaii.* 1989. **28**(7): p. 1883-1886.

9. Hikino, H., et al., *Structure of α-rotunol and β-rotunol.* 1969(32): p. 2741-2742.

10. Hikino, H., et al., *Structure of sugetriol.* 1967. **15**(9): p. 1433-1435.

11. Chhabra, B., et al., *Three new sesquiterpenes from nutgrass (Cyperus rotundus L.) and their evaluation as plant growth regulators.* 2002. **6**: p. 57-59.

12. HiKiNo, H., et al., *Biochemical Syntheses. II. Microbial Transformation of Cyperotundone to Sugeonol and Isopatchoul-4-en-3-on-8α-ol.* 1968. **16**(6): p. 1088-1090.

13. Xu, Y., et al., *Complete assignments of (1)H and (13)C NMR data for two new sesquiterpenes from Cyperus rotundus L.* Magn Reson Chem, 2009. **47**(6): p. 527-31.

14. Kapadia, V.H., et al., *Sesquiterpenoids from the essential oil of Cyperus rotundus.* 1967. **8**(47): p. 4661-4667.

15. Ha, J.-H., et al., *Modulation of radioligand binding to the GABAA-benzodiazepine receptor complex by a new component from Cyperus rotundus.* 2002. **25**(1): p. 128-130.

16. Iwamura, J., et al., *CONSTITUENTS OF ESSENTIAL OILS OF CYPERUS-SEROTINUS ROTTB AND CYPERUS-ROTUNDUS L.* 1977(7): p. 1018-1020.

17. HIKINO, H., et al., *Structure and absolute configuration of kobusone and isokobusone.* 1969. **17**(7).

18. Kalsi, P., et al., *Biogenetically important sesquiterpenes from Cyperus rotundus.* 1995. **66**(1).

19. Kapadia, V., et al., *Structure of mustakone and copaene.* 1963. **4**(28): p. 1933-1939.

20. Paknikar, S., O. Motl, and K.J.T.L. Chakravarti, *Structures of rotundene and rotundenol.* 1977. **18**(24): p. 2121-2124.

21. Koichiro, et al., *Plant-Growth Inhibitors in Purple Nutsedge (Cyperus rotundus L.).* 1980.

22. Hikino, H., et al., *Structure of cyperolone.* 1966. **14**(12): p. 1439-1441.

23. Xu, Y., et al., *Norcyperone, a novel skeleton norsesquiterpene from Cyperus rotundus L.* Molecules, 2008. **13**(10): p. 2474-81.

24. Wang, Q., et al., *A New Eudesmane － type Sesquiterpene of Cyperusrotundus L．.* 2020(5): p. 6.

25. Xu, H.-B., et al., *Chemical structure of cyperotundic acid from rhizomes of Cyperus rotundus.* 2016. **41**(6): p. 4.

26. Park, Y.J., et al., *Sesquiterpenes from Cyperus rotundus and 4alpha,5alpha-oxidoeudesm-11-en-3-one as a potential selective estrogen receptor modulator.* Biomed Pharmacother, 2019. **109**: p. 1313-1318.

27. Zhou, Z., et al., *A new iridoid glycoside and potential MRB inhibitory activity of isolated compounds from the rhizomes of Cyperus rotundus L.* Nat Prod Res, 2013. **27**(19): p. 1732-6.

28. Mohamed-Ibrahim, S.R., et al., *Anti-inflammatory terpenoids from Cyperus rotundus rhizomes.* 2018. **31**(4(Supplementary)): p. 1449-1456.

29. Xu, H.B., et al., *Bioactivity-guided isolation of anti-hepatitis B virus active sesquiterpenoids from the traditional Chinese medicine: Rhizomes of Cyperus rotundus.* J Ethnopharmacol, 2015. **171**: p. 131-40.

30. Zhou, Z.-l., et al., *New iridoid glycosides with antidepressant activity isolated from Cyperus rotundus.* 2016. **64**(1): p. 73-77.

31. Yang, J.L. and Y.P. Shi, *Structurally diverse terpenoids from the rhizomes of Cyperus rotundus L.* Planta Med, 2012. **78**(1): p. 59-64.

32. Wang, Q., et al., *Research on chemical constituents of Cyperus rotundus L.* 2021.

33. Singh, N. and P.J.J.o.t.I.C.S. Singh, *A NEW FLAVONOL GLYCOSIDE FROM THE MATURE TUBERS OF CYPERUS-ROTUNDUS L*. 1986, INDIAN CHEMICAL SOC 92 ACHARYA PRAFULLA CHANDRA RD ATTN: DR INDRAJIT KAR …. p. 450-450.

34. Ueki, K., et al., *Studies on Polyphenolic Substances in Tubers of Eleocharis kuroguwai Ohwi, Cyperus serotinus Rottb. and Cyperus rotundus L.* 1974. **1974**(17): p. 20-24.

35. Sayed, H.M., et al., *Phytochemical and biological investigations of Cyperus rotundus L.* 2001. **39**(3): p. 195-203.

36. Xu, Y., Z.Z. Zhang, and Z.M.J.J.o.C.P.S. Zou, *Studies on chemical constituents of the rhizomes of Cyperus rotundus L.* 2010. **45**(11): p. 818-820.

37. Zhou, Z.L., et al., *Chemical constituents from rhizomes of Cyperus rotundus.* 2013. **44**(10): p. 1226-1230.

38. Wang, Y.J., X.U. Jie, and L.I.J.S.J.o.T.C.M. Yong, *Research on separation, identification and bioactivity of flavonoids in rhizoma cyperi.* 2018. **34**(8): p. 3.

39. Kim, S.K., et al., *Chemical Components of Cyperus rotundus L. and Inhibitory Effects on Nitric Oxide Production.* 2000.

40. Singh, P. and S.J.P. Singh, *New Saponin from mature tubers of Cyperus rotundus.* 1980.

41. Sayed, H.M., et al., *Phytochemical and biological investigations of Cyperus rotundus L.* 2001.

42. Sayed, H.M., et al., *A new steroid glycoside and furochromones from Cyperus rotundus L.* Nat Prod Res, 2007. **21**(4): p. 343-50.

43. Wu, H., et al., *Extraction of Alkaloids from Cyprus Rotundus and Their Antioxidant Activity in Vitro.* 2019. **048**(011): p. 16-17.

44. Wen, D.T., R. Zhang, and S.Z.J.J.o.P.U.M.S. Chen, *Isolation of chemical constituents from Rhizoma Cyperi and their effects on uterine muscle contraction in non-pregnant rats in vitro.* 2003. **35**(1): p. 2.

45. Asenjo, C.F.J.J.o.P.S., *Some of the constituents of the tuber of coqui (Cyperus rotundus L.). III.The sugars.* 2010. **31**(3): p. 88-89.

46. Luo, S.W., et al., *Chemical constituents from Rhizoma Cyperi.* 2014(2): p. 4.

47. Lin, S.-q., et al., *Cyprotuoside C and Cyprotuoside D, two new cycloartane glycosides from the rhizomes of Cyperus rotundus.* 2017: p. c17-00608.

48. Zhou, Z. and W.J.M. Yin, *Two novel phenolic compounds from the rhizomes of Cyperus rotundus L.* 2012. **17**(11): p. 12636-12641.

49. Wu, Y.B., *Preliminary study on the chemical constituents from the bioactivity parts and on the quality evaluation of Rhizoma Cyperi*. 2009, Chengdu University of Traditional Chinese Medicine.
